# Robot obstacle avoidance optimization by A* and DWA fusion algorithm

**DOI:** 10.1371/journal.pone.0302026

**Published:** 2024-04-29

**Authors:** Peiying Li, Lingjuan Hao, Yanjie Zhao, Jianmin Lu

**Affiliations:** 1 Mechanical and Electrical College, Handan University, Handan, 056005, China; 2 College of Mathematics and Physics, Handan University, Handan, 056005, China; Cyprus International University Faculty of Engineering: Uluslararasi Kibris Universitesi Muhendislik Fakultesi, TURKEY

## Abstract

The current robot path planning methods only use global or local methods, which is difficult to meet the real-time and integrity requirements, and can not avoid dynamic obstacles. Based on this, this study will use the improved A-star global planning algorithm to design a hybrid robot obstacle avoidance path planning algorithm that integrates sliding window local planning methods to solve related problems. Specifically, A-star is optimized by evaluation function, sub node selection mode and path smoothness, and fuzzy control is introduced to optimize the sliding window algorithm. The study conducted algorithm validation on the TurtleBot3 mobile robot, with data sourced from experimental data from a certain college. The results showed that hybrid algorithm enabled the planned path to effectively navigate around dynamic obstacles and reach the target point accurately. When compared with traditional methods, path length reduced by 9.6%, path planning time decreased by 29% with an approximate 26.7% increase in the average speed of the robot. Compared with the traditional methods, the research algorithm has greatly improved in avoiding dynamic obstacles, path planning efficiency, model adaptability and so on, which has important value for relevant research. It can be seen that the algorithm proposed in the study has performance advantages, demonstrating the effectiveness and advantages of robot path planning, and can provide reference for robot obstacle avoidance optimization. Research can complete tasks for robots in practical environments, which has certain reference value for the research of robots in path planning and the development of path obstacle avoidance planning.

## 1. Introduction

At present, relevant research of robot only uses global or local methods, which is difficult to satisfy real-time and integrity in complex and changeable environment. Meanwhile, traditional global path planning algorithm, A-star (A*) algorithm, has problems such as the path curvature of the planning is not continuous enough and search time is too long. Dynamic window approach (DWA) has defects of not adapting to complex environment [1]. Based on this, firstly, the algorithm optimization will be carried out in the evaluation function, sub node selection mode and path smoothness of A* for long search time and unsmooth path. Secondly, fuzzy control will be introduced to optimize the DWA algorithm to improve algorithm adaptability. The main novelty of this study is that it proposes a method that integrates global path planning with local obstacle avoidance strategies. This method provides an optimized initial path for the robot through an efficient global planning algorithm, and combines with an advanced local planning algorithm to dynamically adjust the path to cope with emergencies. This fusion strategy greatly improves the robot’s adaptability and decision-making speed in complex environments, ensuring that the path can still be updated in real time when encountering unforeseen obstacles and avoiding planning failures. This not only enhances the autonomous navigation ability of robots, but also improves the accuracy and efficiency of path planning, which brings new theoretical and practical value to the research field of robot navigation system.

The academic contribution of this research is as follows: By improving ergodic rules and evaluation functions, the performance of traditional path planning algorithm in dealing with emergencies and dynamic environments is optimized, and new ideas and methods are provided for solving path planning problems in practical applications. The path safety and stationarity are further improved. In traditional path recognition algorithms, the distance between robots and obstacles is too small, which may lead to collisions. Therefore, the dynamic obstacle avoidance path planning proposed in this study reduces unnecessary safety risks. Due to the introduction of dynamic window coordinator algorithm, the efficiency of local path planning is accelerated, the efficiency and accuracy of path planning are effectively improved, and the real-time performance and robustness are better when dealing with accidental and sudden environmental changes. Finally, an optimization algorithm combining global path planning and local path planning is studied to achieve a higher traversal rate. Therefore, the research and design of the mobile robot control scheme strengthens the system’s adaptability to the dynamic environment during path planning, especially how to better cope with emergencies. To sum up, the research has made an important academic contribution to the field of dynamic path planning, provided a series of solutions with practical application value, focused on improving the safety, real-time and accuracy of path planning, and provided innovative methods for autonomous navigation of robots in uncertain and changing environments. These contributions will have a positive impact on the future research and practical application of robotics.

Finally, research will design a hybrid method by improved A* and dynamic window, to solve existing robot obstacle avoidance path planning problems. This research is mainly carried out from the following four aspects. The first part introduces the research status of effective obstacle avoidance and path planning; The second part is specific design of robot obstacle avoidance fusion algorithm; The third part is performance and practicability verification through simulation experiments; The fourth part is the summary and analysis of the full text.

## 2. Related works

As intelligent robots develop, many scholars conducted relevant research on their path planning.Q. Li and W. Lin, aiming at the problem that traditional graphical neural network algorithm for robot route planning relies on a simple information aggregation mechanism and cannot prioritize important information, introduced a similar information query mechanism to establish a message aware graphical attention network, so that the robot can judge the importance of feature information from adjacent robots. The simulation results show that the model can be well extended in previously unseen problem instances, and it has increased the success rate by 47% compared to the graphical neural network [2]. AB Wahab Mn team proposed a path planning algorithm combined with heuristic algorithm for poor effectiveness of meta heuristic algorithm in robot coordination agent, and verified the feasibility of this method through simulation experiments. The results showed that this method had better adaptability to unknown environment than traditional methods [3]. J. Qi, H. Yang and H Sun proposed a path planning search algorithm. This method used the modified fast random search tree algorithm to obtain the initial path, and re-planned the path by optimizing the initial path. Results showed that a high performance in avoiding unknown obstacles [4]. B. Wang team proposed a global guided reinforcement learning method to solve low efficiency and long planning path of traditional path planning in dynamic obstacle environment. Simulation experiments were carried out on grid maps. The results showed that the algorithm had better generalization ability [5]. B. B. K. Ayawli team proposed a robot method in complex dynamic environment by computational geometry technology. The method used morphological expansion, A* algorithm and cubic spline algorithm to calculate the initial path, and used Kalman filter algorithm to calculate the new path for re planning. The simulation results showed that success path calculation rate of the method had better performance in path cost and number of re planning calculations [6].

In terms of path planning of A* and DWA, many scholars have carried out relevant research. Xiangrong T team proposed three methods to optimize the algorithm, including two-way search, etc., to solve that A* occupies a large memory space compared with other path planning algorithms, and verified the algorithm feasibility through MATLAB experiments. Results showed that optimization algorithm can reduce the memory occupation by more than 60% [7]. L. Zhang and Y. Zhang considering the disadvantages of poor local planning effect and low overall planning efficiency, proposed a algorithm to prevent algorithm from falling into local minimum by modifying inertia weight and acceleration factor, and improved efficiency by fitness variance optimization algorithm, The feasibility of this method was verified by simulation [8]. Based on improved A* and DWA, Li Y team used lidar to build a map, and proposed a method for jujube orchard dense planting. The method used the hybrid algorithm to achieve global and local path planning, and optimized path through evaluation function. Results showed that it was superior to traditional fusion algorithm [9]. Linz team proposed a method to conduct global and local robot path planning. In terms of local path planning, fuzzy controller was introduced to optimize the DWA algorithm, and path planning method effectiveness was verified [10]. Wang Q team proposed an algorithm to coordinate multi robots and path planning between robots and unknown areas. The algorithm combined ant colony algorithm and DWA, coordinated multi robot system through priority strategy, and obtained the global optimal path through redundant point deletion strategy. Results showed that it achieved cooperative obstacle avoidance, And it had high security and global optimality [11]. To enhance robot ability to plan the overall route in static and dynamic terrain, Vikas and Parhi D R proposed a robot path planning algorithm combining improved hyperbolic gravity search algorithm and dynamic window method, and carried out simulation experiments in static and dynamic terrain. The results showed that its obstacle avoidance effect was better than other sensor based methods [12].

S. The Wang team proposed an adaptive obstacle avoidance algorithm based on DDPG (Deep Deterministic Policy Gradient) and DWA (Dynamic Window Approach) for studying the obstacle avoidance problem of robots in complex continuous state spaces. The experiment shows that the model can significantly overcome the limitation of DWA algorithm to local optimal solutions in complex environments; Through trial and error interaction with the environment and trajectory evaluation feedback, the obstacle avoidance ability of robots in complex environments has been improved [13]. X. Zhang et al. analyzed the applicability of three SLAM algorithms in indoor rescue environments by combining path planning algorithms. In order to balance path optimization and obstacle avoidance, the A * algorithm is used for global path planning, and the DWA algorithm is used for local path planning. The results indicate that studying algorithms can help researchers quickly and clearly select suitable algorithms to build SLAM systems based on their own needs [14]. Mobile robots face more challenging issues in real-time path planning and collision free path tracking when used in large-scale dynamic environments. X. Zhong and his team proposed a new hybrid path planning method that combines the a * algorithm with the adaptive window method to perform global path planning, real-time tracking, and obstacle avoidance for mobile robots in large dynamic environments. The results indicate that the proposed hybrid path planning method is suitable for global path planning, tracking, and obstacle avoidance, and can meet the application requirements of mobile robots in complex dynamic environments [15].

To sum up, at present, most of the research is carried out in static environment, which only adopts global or local methods. In complex and changeable environment, it is difficult to satisfy real-time and integrity. Meanwhile, the fusion of A* and DWA has some defects, such as not suitable for complex environment. Therefore, this research will integrate improved A* and DWA design model for mobile robot path planning to improve planning efficiency and dynamic environment adaptability.

## 3. Robot obstacle avoidance path planning with A* and DWA fusion algorithm

Traditional research only uses global or local path planning methods, which is difficult to satisfy real-time and integrity in the application. This research will design a hybrid path planning with improved A* and DWA for path planning improvement.

### 3.1. Improved A* global path planning algorithm design

**Fig 1:**
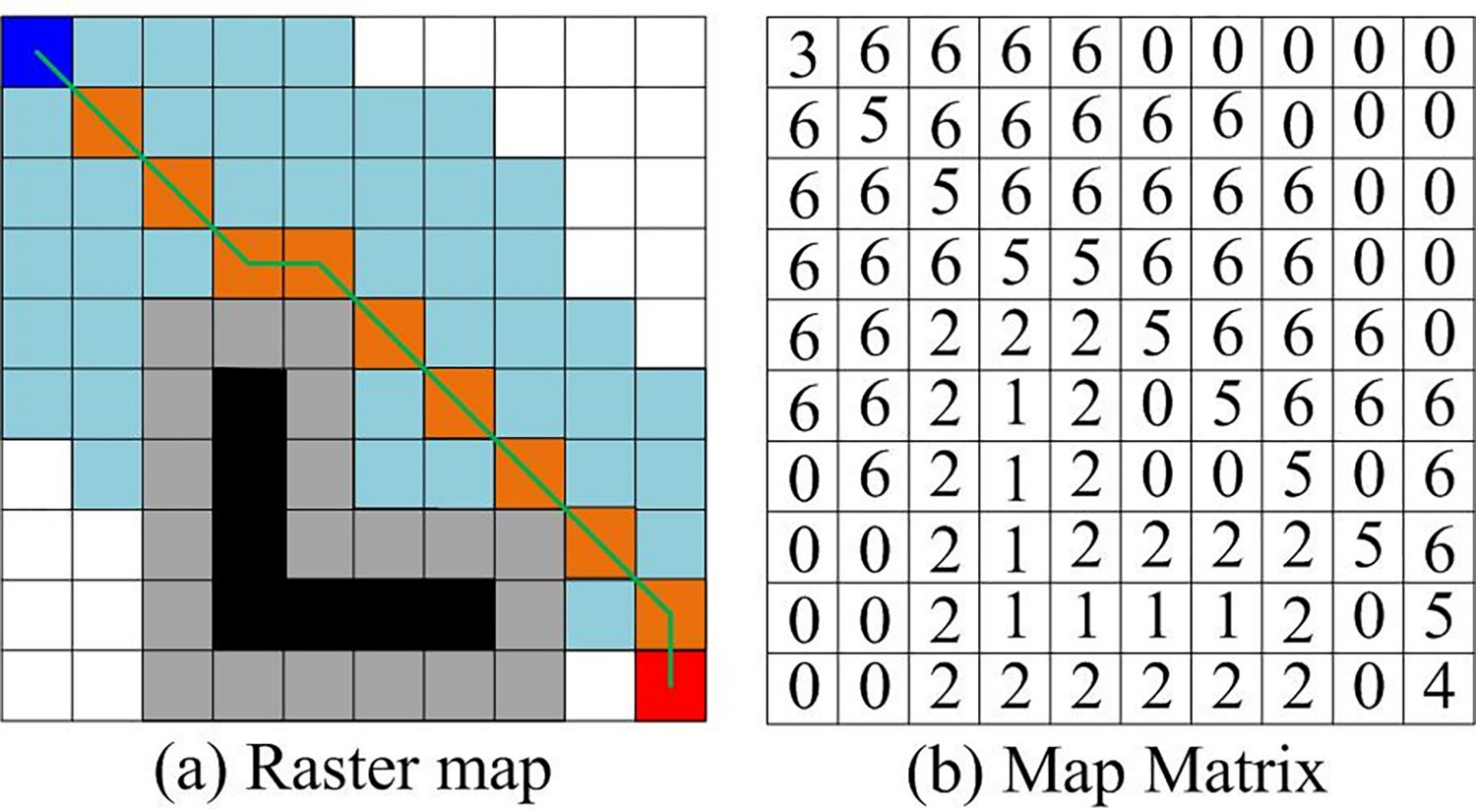
Basic structure of extended revolutionary kernel.

Traditional A* considers the scale of the robot itself as a particle, which will cause the path trajectory to cling to obstacle and increase collision probability [16]. Research will generate a layer of extended area around the obstacle to maintain a certain distance between path and obstacleto obtain a better obstacle avoidance effect. The grid map and extended area are shown in Fig 1.

In Fig 1, mobile robot is regarded as a 1*1 square. The map expresses robot’s environmental information. The robot’s working environment is divided into seven states and represented by 0–6 in the corresponding matrix. Among them, the white grid represents the range that the mobile robot passes through (No. 0), the black grid represents obstacles that cannot be passed through (No. 1), the gray grid represents the expanded range of obstacles (No. 2), the blue grid represents the starting point (No. 3), the red grid represents the target point (No. 4), the orange grid represents the target point (No. 5), and the light blue grid represents the places that the robot has searched (No. 6). When using A* for path planning, it is necessary to set obstacle rate according to the grid map to express the distribution of obstacles. Obstacle rate is calculated in Formula (1).


$$P=\frac{N}{\left(|x_{s}-x_{g}|+1)\times (|y_{s}-y_{g}|+1\right)}(P\in (0,1))$$ (1)

As shown in Formula (1), *P* represents the obstacle rate, *N* counts obstacle grids, (*x*_*s*_,*y*_*s*_) is the coordinate of starting node, and (*x*_*g*_,*y*_*g*_) is that of target node. Manhattan distance, also known as urban block distance, is the sum of distances along the grid direction between two points. Compared to the Euclidean distance, the Manhattan distance obtained in robot path planning is generally not the shortest distance. Therefore, the study drew inspiration from Euclidean distance. The research on the improved A* algorithm will first improve the evaluation function, introduce the obstruction rate into evaluation function, and make it have strong self-adaptive ability and global optimization ability according to the adjusted weights of *g*(*n*) and *h*(*n*) [17]. Formula (2) shows its function.


f(n)=g(n)+(1−lnP)h(n) (2)

As shown in Formula (2), *f*(*n*) represents (*x*_*n*_,*y*_*n*_) evaluation function, *h*(*n*) represents proxy value, *g*(*n*) represents estimated cost from current (*x*_*n*_,*y*_*n*_) to target (*x*_*s*_,*y*_*s*_), and represents mobile cost from starting to current. Formula (3) shows the mathematical expression formula of *g*(*n*).


$$g(n)=\sum_{\left(x_{n},y_{n})=(x_{s},y_{s}\right)}^{\left(x_{g},y_{g}\right)}\sqrt{{\left(x_{n}-x_{n-1}\right)}^{2}+{\left(y_{n}-y_{n-1}\right)}^{2}}$$ (3)

The calculation formula of *h*(*n*) is shown in Formula (4).


$$h(n)=\sqrt{{\left(x_{n}-x_{g}\right)}^{2}+{\left(y_{n}-y_{g}\right)}^{2}}$$ (4)

**Fig 2:**
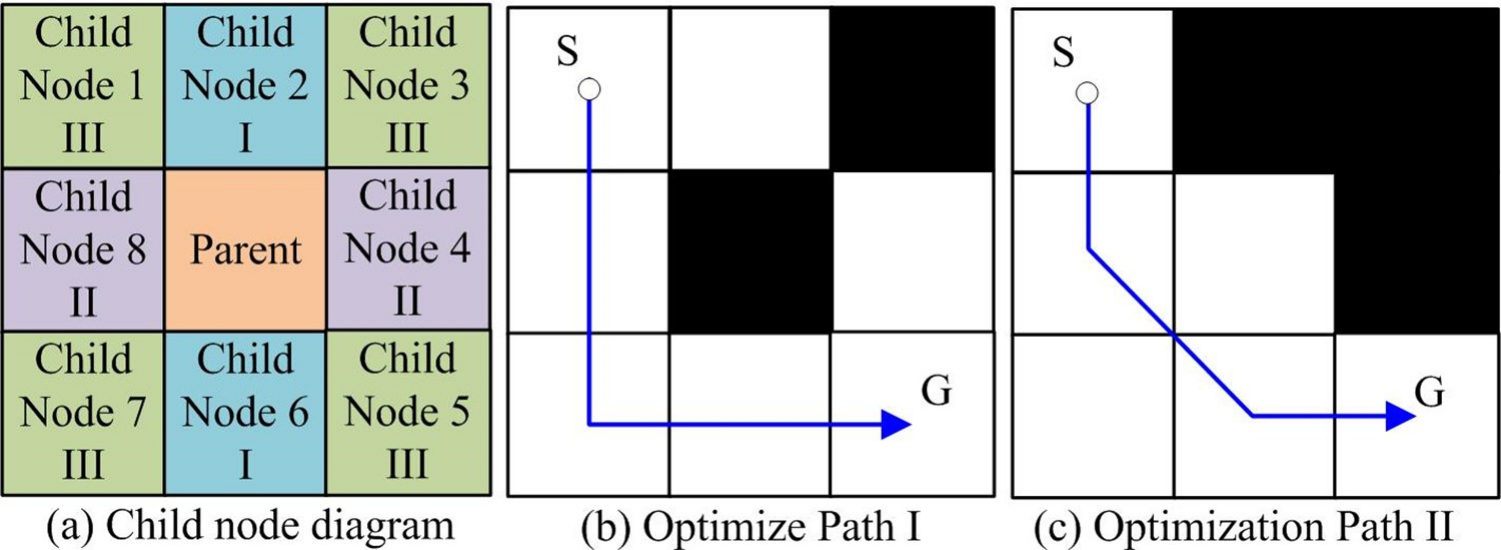
Schematic diagram of optimizing sub nodes.

Traditional A* algorithm path planning method directly plans the path by judging the smallest node through the evaluation function. There is a problem that path may pass through obstacle vertex and is prone to collision. This study will optimize the selection of child nodes through the position relationship between parent nodes and child nodes. The position relationship of each child node and the corresponding optimized path are shown in Fig 2.

**Fig 3:**
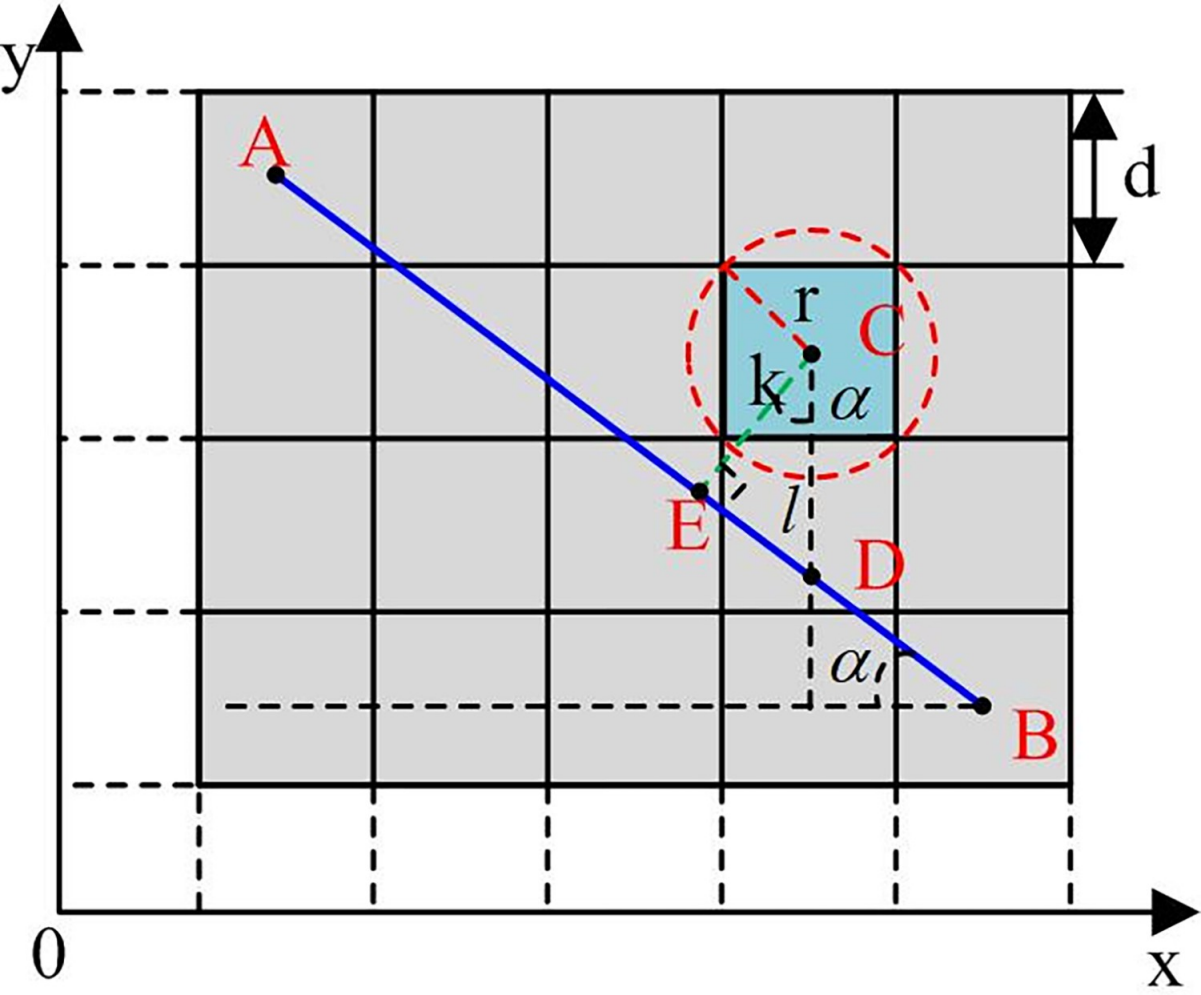
Schematic diagram of path smoothing distance relationship.

As shown in Fig 2(A), after the parent node generates child nodes, the child nodes are divided into three types based on the position relationship. Type I is the upper and lower child nodes, type II is the left and right child nodes, and type III is remaining child nodes. In Fig 2(B) and 2(C) optimization path diagram, when obstacle is in type III, no processing is done. When the obstacle is in the class I and II positions, the left and right or upper and lower optional nodes of the obstacle are deleted to prevent the path from passing through the vertex of the obstacle. Traditional A* has too many turns, poor smoothness and non shortest path, because its path node is only located in the center of the grid. To solve them, bi-directional smoothness of path nodes is optimized in safe distance selection to avoid collision. The distance relationship of bi-directional smoothness optimization criteria is shown in Fig 3.

In Fig 3, for the node of any path between A and B, C is the center node of the obstacle, cellside length is set to *d*, and CD to l, indicating the longitudinal distance from C to AB; CE length is set to *k*, indicating the vertical distance from point C to AB; *α* represents the angle between AB and X axis, and *r* represents circumscribed obstacle radius at point C. The safety distance is *K*, and *K*≥*r* is satisfied, *k*≤*K*, then the robot will hit an obstacle, indicating that the path is not optional; If *k*≥*K*, it is a safe path and can be selected. The calculation formula of *k* is shown in Formula (5).


k=lcosα (5)

As shown in Formula (5), the specific mathematical calculation formula of *l* is shown in Formula (6).


$$l=|y_{c}-[\frac{y_{a}-y_{b}}{x_{a}-x_{b}}(x_{c}-x_{a})+y_{a}]|$$ (6)

As shown in Formula (6), (*x*_*a*_,*y*_*a*_), (*x*_*b*_,*y*_*b*_), and (*x*_*c*_,*y*_*c*_) are coordinates of A, B, and C. The coordinate values of *x*_*a*_ and *x*_*b*_ are different, *x*_*a*_ ≠ *x*_*b*_. *α* calculation formula is shown in Formula (7).


$$\alpha =|\arctan\frac{y_{a}-y_{b}}{x_{a}-x_{b}}|$$ (7)

**Fig 4:**
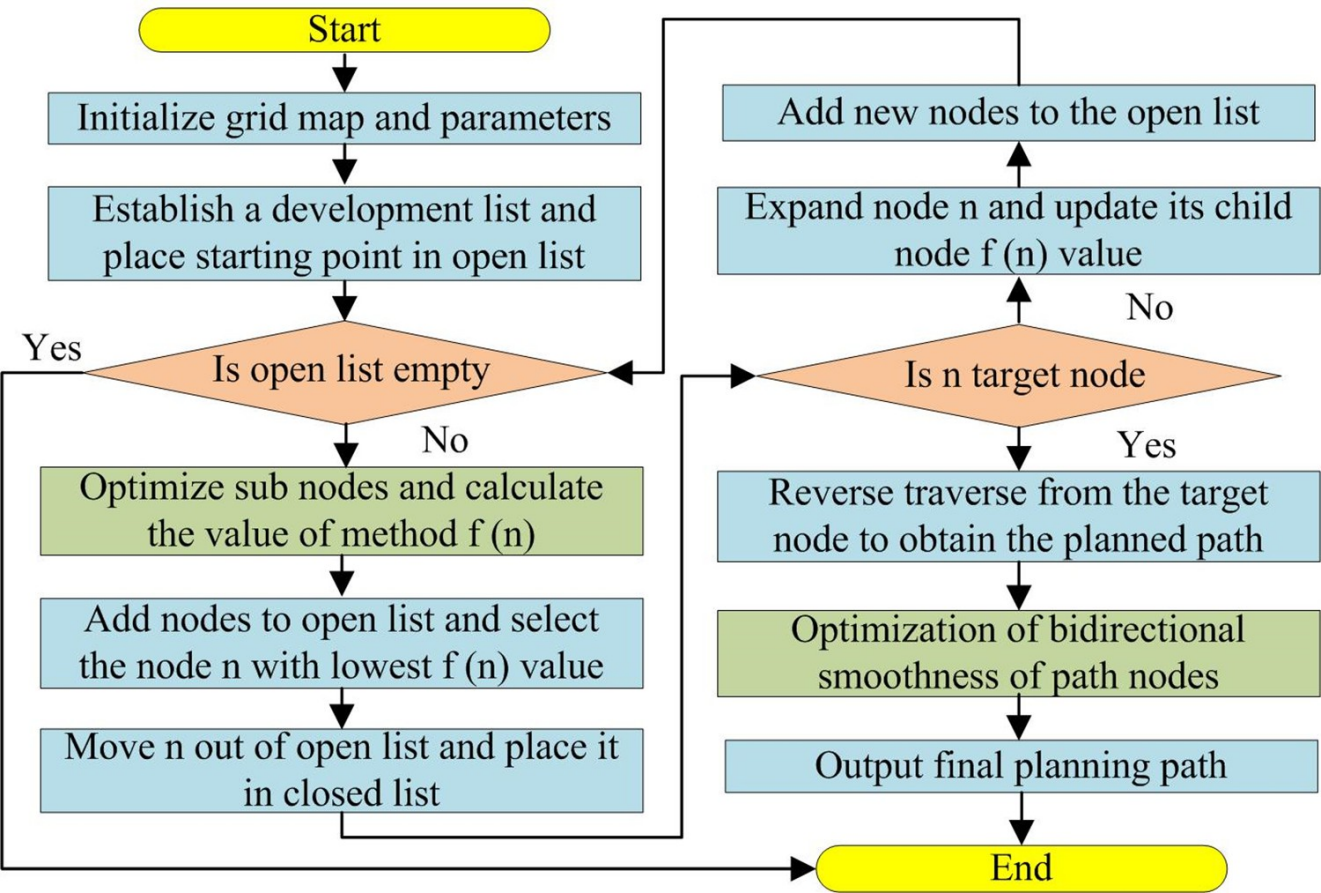
Improved A* algorithm flowchart.

This method optimizes the bi-directional smoothness of the path from two nodes a and B, and adds the obstacle distance criterion, so that the path node can choose anywhere in the grid,to obtain a shorter and smoother path with fewer path turns. The research establishes a path planning model for improved A*in Fig 4.

In Fig 4, firstly, avoidance environment is modeled by the optimized grid method; Secondly, starting and target points are set, then open and closed lists are set, starting point is put in open list. If open list is empty, there are no nodes, and the end algorithm does not generate planning path; Then, algorithm starts to traverse surrounding nodes near starting point, and according to optimized sub node selection method, selects node with the smallest f (n) as new node, and repeats traversal from judging open list until target node is found; Finally, planning path is obtained by traversing target node of the path in reverse, and the bi-directional smoothness of the path node is optimized to obtain and output the final global planning path.

### 3.2. Robot obstacle avoidance algorithm with DWA

**Fig 5:**
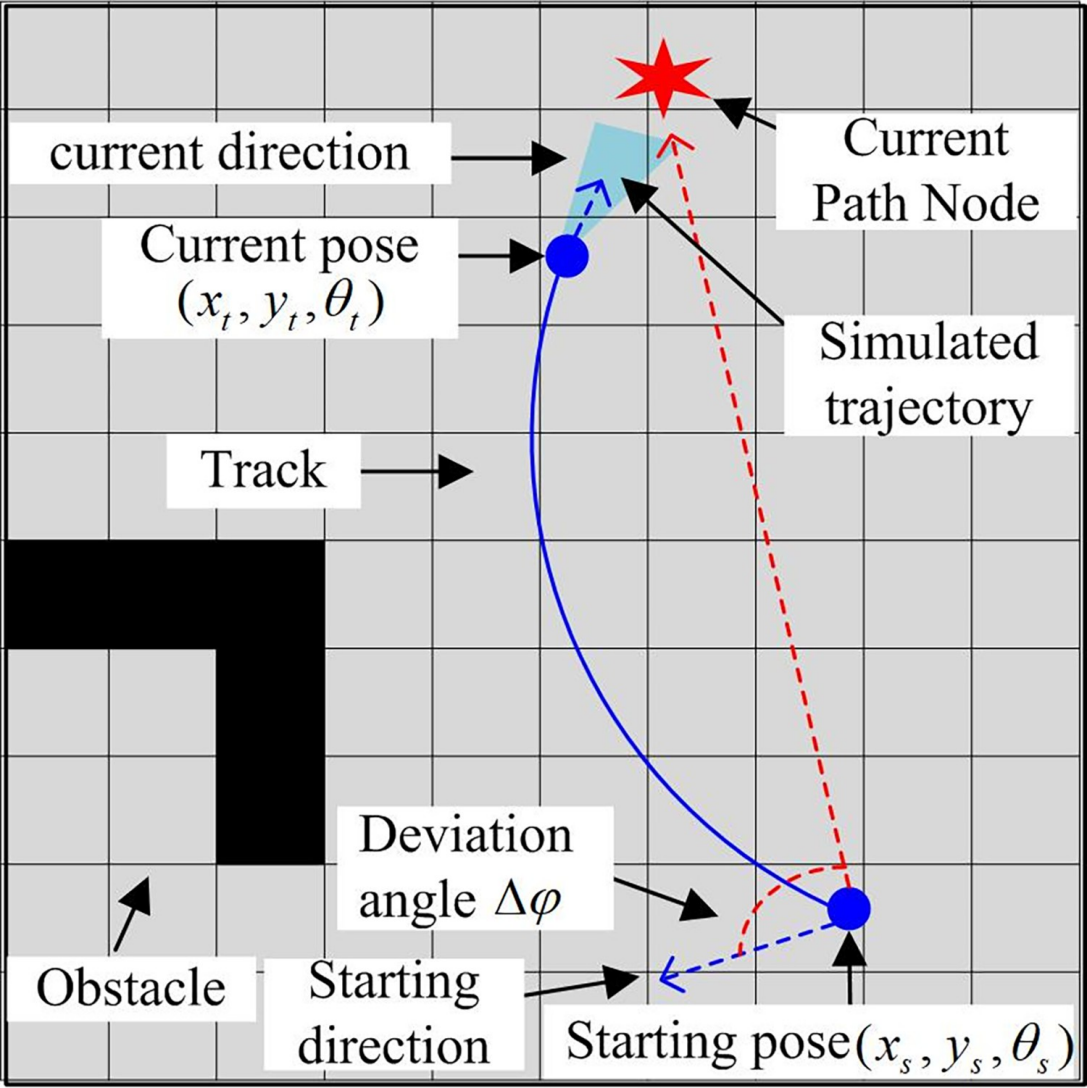
Improved A* flowchart.

The improved A* can improve path planning in the static environment. However, in situations where there are uncertain and dynamic obstacles during the planning process, if the mobile robot continues to follow globally planned path, it will be unable to avoid these obstacles [18]. Therefore, this research will introduce DWA-based path planning to optimize robot mobile obstacle avoidance, and optimize local path planning effect of algorithm by improving DWA, so that robot can better achieve dynamic obstacle avoidance. First of all, the path planning with traditional DWA, which only takes target position as the reference direction, will lead to the problems that the robot exists in the actual movement, the initial pose and the current path pose direction have deviation, the turning amplitude becomes larger, and the algorithm efficiency decreases. The attitude adjustment function is introduced to optimize the algorithm and eliminate the attitude deviation. Fig 5 shows the movement trajectory and attitude direction.

Δ*φ* in Fig 5 shows angular deviation between robot attitude direction at starting point and the current path node, which will affect path planning length. Larger value represents greater path turning range, resulting in longer path planning and lower efficiency. The research will add a posture adjustment function, so that the algorithm can adjust the initial posture of the robot before local path planning, so that it is consistent with each node in the current path. Formula (8) shows the specific expression formula of posture adjustment function.


$$\Delta \varphi =\frac{1}{2}\dot{w}t^{2}(\dot{w}\in [{\dot{w}}_{\min},{\dot{w}}_{\max}])$$ (8)

As shown in Formula (8), $\dot{w}$ represents the angular acceleration of the starting position of the robot, *t* is the attitude adjustment time, ${\dot{w}}_{\min}$ is the minimum acceleration, and ${\dot{w}}_{\max}$ is the maximum acceleration. Smaller *d* means robot is closer to obstacle, and the score is lower. DWA algorithm needs to sample a small speed before further planning robot obstacle avoidance path. There are three types of speed sampling constraints for the algorithm. The first type is based on kinematic constraints, and the relevant mathematical expression formula is shown in Formula (9).


$$V_{s}=\{\left(v,w),v\in [v_{\min},v_{\max}]\wedge w\in [w_{\min},w_{\max}\right]\}$$ (9)

As shown in Formula (9), *V*_*s*_ represents the maximum and minimum speed limits, *v* is linear speed, *w* represents angular speed, *v*_max_ and *v*_min_ represent maximum and minimum linear speed, *w*_max_ and *w*_min_ represent maximum and minimum angular speed. The second type is based on constraints related to motor dynamics, and the relevant mathematical expression formula is shown in Formula (10).


$$V_{d}=\{\left(v,w),v\in [v_{c}-{\dot{v}}_{b}\Delta t,{\dot{v}}_{a}\Delta t]\wedge w\in [w_{c}-{\dot{w}}_{b}\Delta t,{\dot{w}}_{a}\Delta t\right]\}$$ (10)

As shown in Formula (10), *V*_*d*_ represents the motor speed limit, *v*_*c*_ and *w*_*c*_ represent actual linear and angular velocity at any time *t*, ${\dot{v}}_{a}$ and ${\dot{v}}_{b}$ represent the maximum acceleration and deceleration of actual linear velocity, ${\dot{w}}_{a}$ and ${\dot{w}}_{b}$ represent those of the actual angular velocity.


$$V_{a}=\{(v,w)|v\leq \sqrt{2\cdot dest(v,w)\cdot {\dot{v}}_{b}})\wedge w\in \sqrt{2\cdot dest(v,w)\cdot {\dot{w}}_{b}}\}$$ (11)

**Fig 6:**
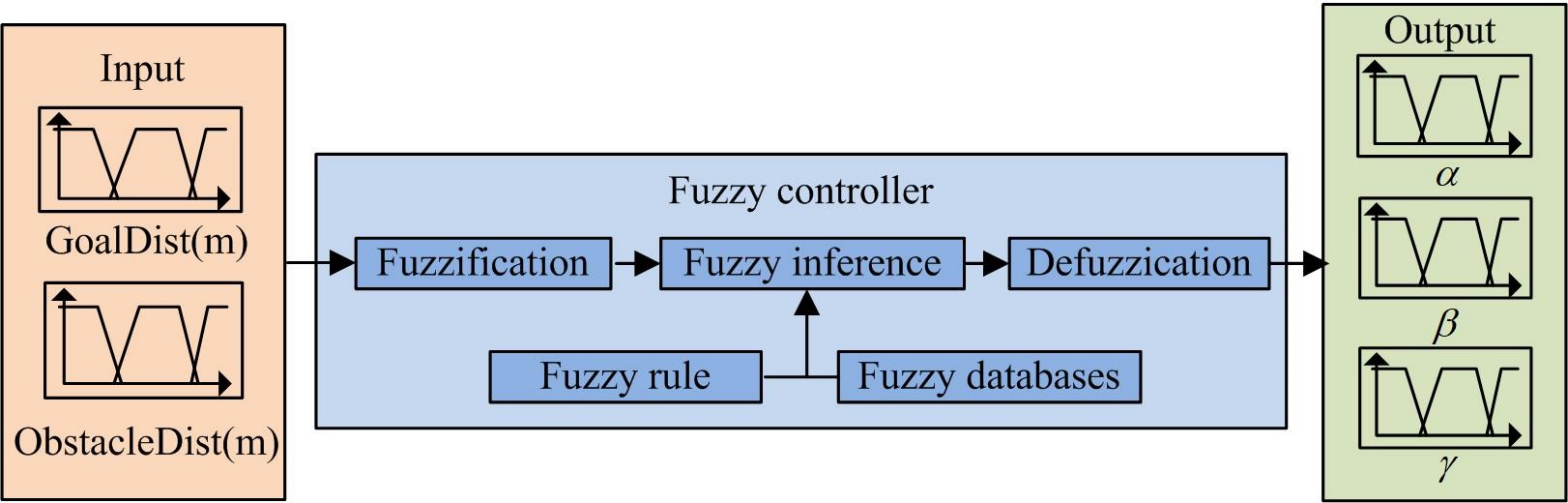
Structure diagram of fuzzy controller.

As shown in Formula (11), *V*_*a*_ represents the speed limit at the moment before the collision, and *dest*(*v*,*w*) represents shortest distance between current (*v*,*w*) corresponding trajectory and the obstacle. At the same time, the traditional DWA algorithm uses fixed weight combination in different environments, which is easy to cause problems such as obstacle avoidance, path length increase, and target point loss. Based on the DWA algorithm, this research will introduce a fuzzy controller to adjust trajectory evaluation function weight in real time to enhance adaptive ability of algorithm. Fig 6 shows DWA fuzzy controller structure.

The input GoalDist in Fig 6 is the membership distance function from robot to target point, which can increase robot flexibility to avoid obstacles to distant targets. The input ObstacleDist avoids missing targets due to being close to the target node [19]. The output is the weighting coefficient *α*, *β*, and *γ*. At the same time, the research will also optimize DWA evaluation function by adding weight *λ*, and distinguish obstacles by adding the azimuth angle of sub target points, so as to avoid obstacles. Formula (12) shows the improved evaluation function.


G(v,w)=α⋅head(v,w)+β⋅vel(v,w)+γ⋅dist_sta(v,w)+λ⋅dist_dyna(v,w) (12)

In Formula (12), *α*, *β*, *γ*, and *λ* are the weighting coefficients, *head*(*v*,*w*) represents azimuth, *vel*(*v*,*w*) represents current acceleration value, *dist*_*sta*(*v*,*w*) represents the shortest distance between planned path and static obstacle, and *dist*_*dyna*(*v*,*w*) represents the shortest distance between planned path and dynamic obstacle. Formula (13) shows the mathematical expression formula of *head*(*v*,*w*).


$$head(v,w)=\frac{\pi -\Delta \varphi_{i}}{\sum_{i=1}^{N}\left(\pi -\Delta \varphi_{i}\right)}$$ (13)

As shown in Formula (13), *N* counts robot simulated trajectories, *i* represents the *i* track, and Δ*φ*_*i*_ represents the key attitude azimuth deviation of the *i* track. The closer the robot is to the end point, the smaller the Δ*φ* value, the higher the proportion of azimuth, and the higher the score of the algorithm. The mathematical expression formula of *vel*(*v*,*w*) is shown in Formula (14).


$$vel(v,w)=\frac{v_{i}}{\sum_{i=1}^{N}v_{i}}$$ (14)

In Formula (14), *v*_*i*_ represents the acceleration value at the end point of the *i* track. Greater *vel*(*v*,*w*) proportion means faster speed and higher score. The expression formula of the nearest distance *dist*(*v*,*w*) between the robot and the obstacle is shown in Formula (15).


$$dist(v,w)=\frac{d_{i}}{\sum_{i=1}^{N}d_{i}}(d_{i}=\{\begin{array}{l} d_{i},d_{i}<D_{o}\\ D_{o},d_{i}\geq D_{o} \end{array})$$ (15)

**Fig 7:**
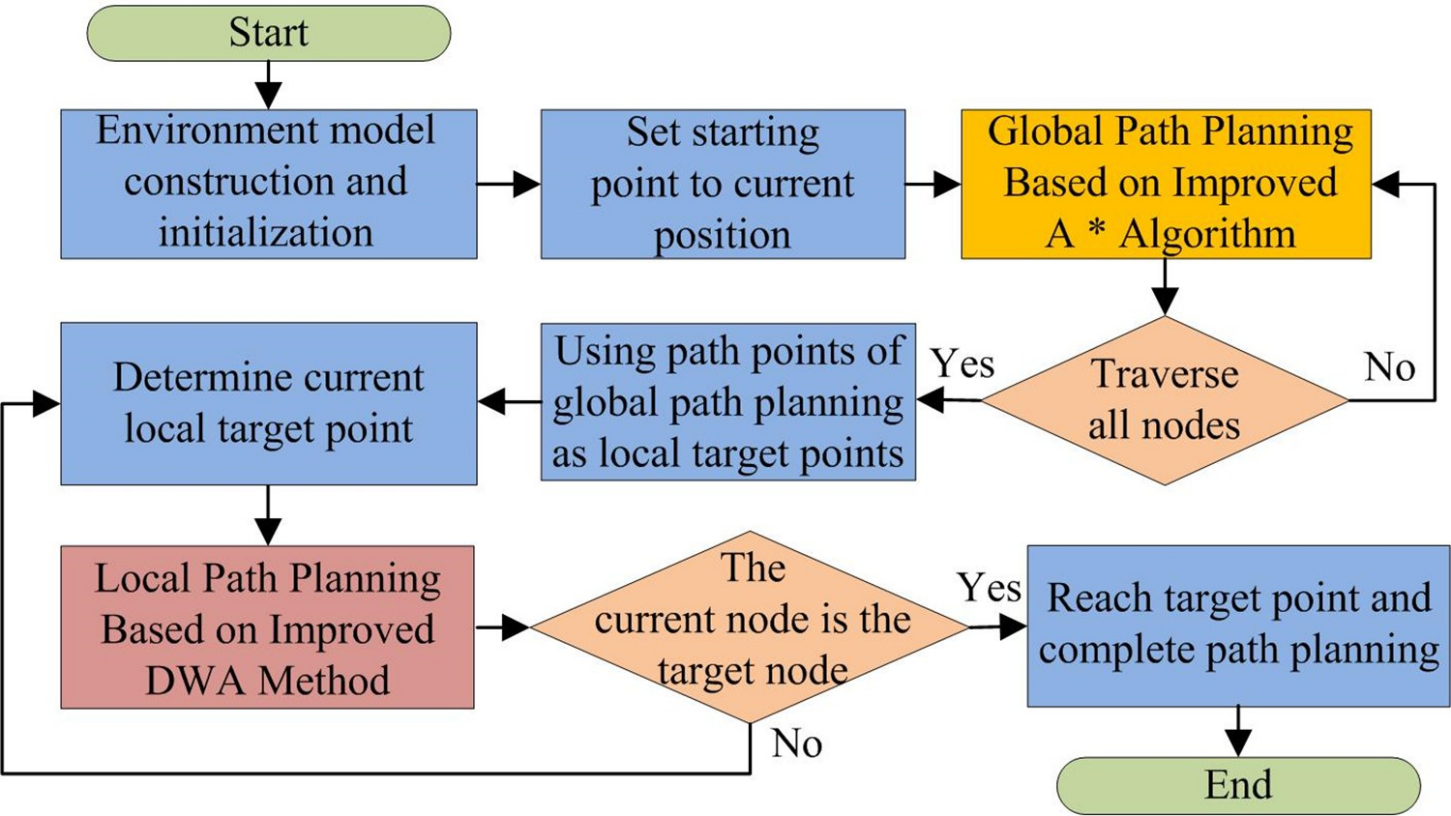
Path planning flowchart for improving A* and DWA fusion algorithms.

As shown in Formula (15), *D*_*o*_ is a constant set by itself and *d*_*i*_ is the shortest distance from end point of *i* track to obstacle. When obstacle state is known static, the value *dist*(*v*,*w*) is assigned to *dist*_*sta*(*v*,*w*); when the obstacle is dynamic unknown, the value *dist*(*v*,*w*) is assigned to *dist*_*dyna*(*v*,*w*). After the global route design is completed, the global optimal node sequence is generated, and the DWA algorithm needs to continuously make local optimal choices among adjacent nodes, and use the attitude adjustment function to solve the problem of excessive deviation between the initial pose and the current path pose direction, and finally achieve dynamic obstacle avoidance. Improved A* and DWA are combined to design model of hybrid obstacle avoidance path planning. Fig 7 showspath planning process of hybrid algorithm model.

As shown in Fig 7, first, the model obtains the environmental information, and models the obstacle avoidance environment through optimized grid method. Second, it determines that the robot is starting and target node, traverses all target points through improved A*, and selects static global optimal path through global path planning. Then, improved DWA obtains local information, avoid dynamic and unknown obstacles, and judge whether the robot reaches target node. If it reaches it, the algorithm is ended. Otherwise, local path planning is repeated. That is, model first uses improved A* to plan optimal path, and then determines local information according to improved DWA to plan local obstacle avoidance path and obtain final optimal obstacle avoidance path.

## 4. Robot obstacle avoidance path planning experimental results analysis with A* and DWA fusion algorithm

This research will carry out simulation experiments on improved A*, improved DWA and hybrid obstacle avoidance on the MATLAB platform, and introduce relevant algorithms for comparative experiments to verify research algorithm performance. Meanwhile, research will carry out actual obstacle avoidance path planning experiments on the TurtleBot3 mobile robot to verify algorithm feasibility in practical application [20].

### 4.1. Obstacle avoidance path planning simulation results analysis with improved A*

Experiment will be simulated on the MATLAB platform. In order to further verify the reliability of the proposed A*-DWA algorithm, HFAMCPSO and RRT*-PSO algorithms were introduced for control experiments. The HFAMCPSO algorithm is an adaptive mutation chaotic particle swarm optimization algorithm. The RRT *—PSO algorithm is a new path planning algorithm that utilizes the advantage of fast exploration speed of random trees. In the environment grid map, blue triangle represents robot starting point, red square represents target point, blue dot represents current node, black grid represents static obstacles, purple box represents the dynamic obstacles, white grid represents movable area, and side length of each grid is set to 1m. Before the experiment, the evaluation function parameters, related speed and time parameters of the DWA algorithm were set as shown in Table 1.

**Table 1:** Parameter setting.

Parameter name	Numerical value	Parameter name	Numerical value
Target ambiguity evaluation function weight α	0.8	Minimum angular velocity wmin	-20deg/s
Weight of observer clearance evaluation function β	0.1	Minimum linear acceleration’av	0
Speed evaluation function weight γ	0.1	Minimum angular acceleration’aw	-50.0deg/s2
Maximum linear speed Vmax	2.0m/s	Linear velocity resolution	0.01m/s
Maximum angular velocity Wmax	20.0deg/s	Angular velocity resolution	1.0deg/s
Maximum linear acceleration AV	0.2m/s2	Forecast period	3.0s
Maximum angular acceleration aw	50.0deg/s2	Data interval	0.1s
Minimum linear speed Vmin	0	/	/

Firstly, improved A* is simulated and compared with traditional A*. The performance of the algorithm is analyzed by taking the smooth path length, path turning times and robot search time as parameters. At the same time, to better compare their performance and remove accidental factors influence, four different sizes of grid maps were constructed for simulation experiments, and the selected grid sizes were 10 × 10. 30 × 30, 50 × 50 and 100 × The specific experimental results are shown below.

As shown in Table 2, at 10 ×10 grid map of improved traditional A*, smoothed path length is reduced by about 17%, planning time decreases by nearly 18%, and the number of turns decreases by about 55%. At 30 ×30 grid map, the smoothed path length decreases by about 16%, path planning time decreases nearly 21%, and the number of turns decreases by about 43%. At 50 ×50 grid map, the smoothed path length is reduced by about 15%, path planning time decreases by nearly 26%, and the number of turns decreases by about 38%. At 100 ×100 grid map, smoothed path length decreases by about 12%, path planning time decreases by nearly 30%, and the number of turns is reduced by about 22%. The results show that with the increase of the size of the grid map, the reduction of path planning time of improved algorithm is also increasing. At the same time, improved algorithm has obvious improvements in the smooth path length, etc. Parameter testing is a method of inferring the parameters of the population distribution, such as mean and variance, when the form of the population distribution is known. However, people often cannot make simple assumptions about the overall distribution pattern, and parameter testing methods are no longer applicable. Non parametric tests are a type of method based on this consideration, which infers the distribution pattern of the population using sample data when the population variance is unknown or known very little. Due to the fact that non parametric testing methods do not involve parameters related to population distribution in the inference process, they are named "non parametric" testing. Therefore, statistical analysis was conducted on the path "—" using non parametric hypothesis testing and inference analysis. To verify algorithm performance, two kinds of common path algorithms, genetic algorithm (GA) and simulated annealing algorithm (SA) [21,22], are introduced to conduct comparative experiments. In the grid map of 30× 30 and 40 × 40, simulation experiments were carried out below.

**Table 2:** Algorithm simulation results in different size environments.

Environment map	Algorithms	Smoothed path length/m	Planned time/ms	Number of turns
ten × ten	A*	27.97	18.64	11
	Research improvement A*	23.34	15.31	5
thirty × thirty	A*	45.21	37.91	16
	Research improvement A*	37.97	32.12	9
fifty × fifty	A*	77.37	99.37	34
	Research improvement A*	65.36	73.28	21
one hundred × one hundred	A*	152.87	332.73	76
	Research improvement A*	135.28	231.38	59

**Fig 8:**
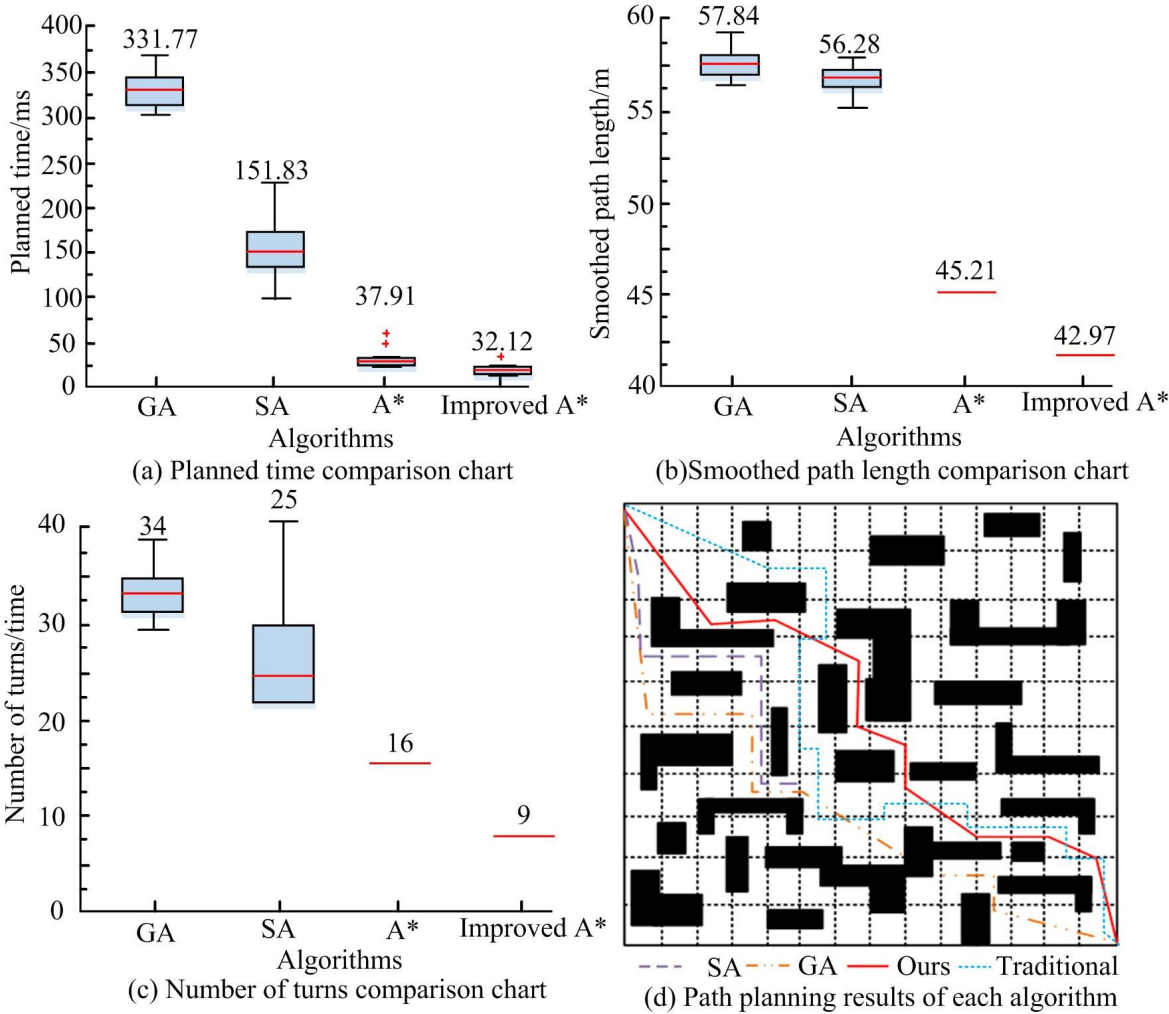
Box plot of experimental results for different algorithms.

**Fig 9:**
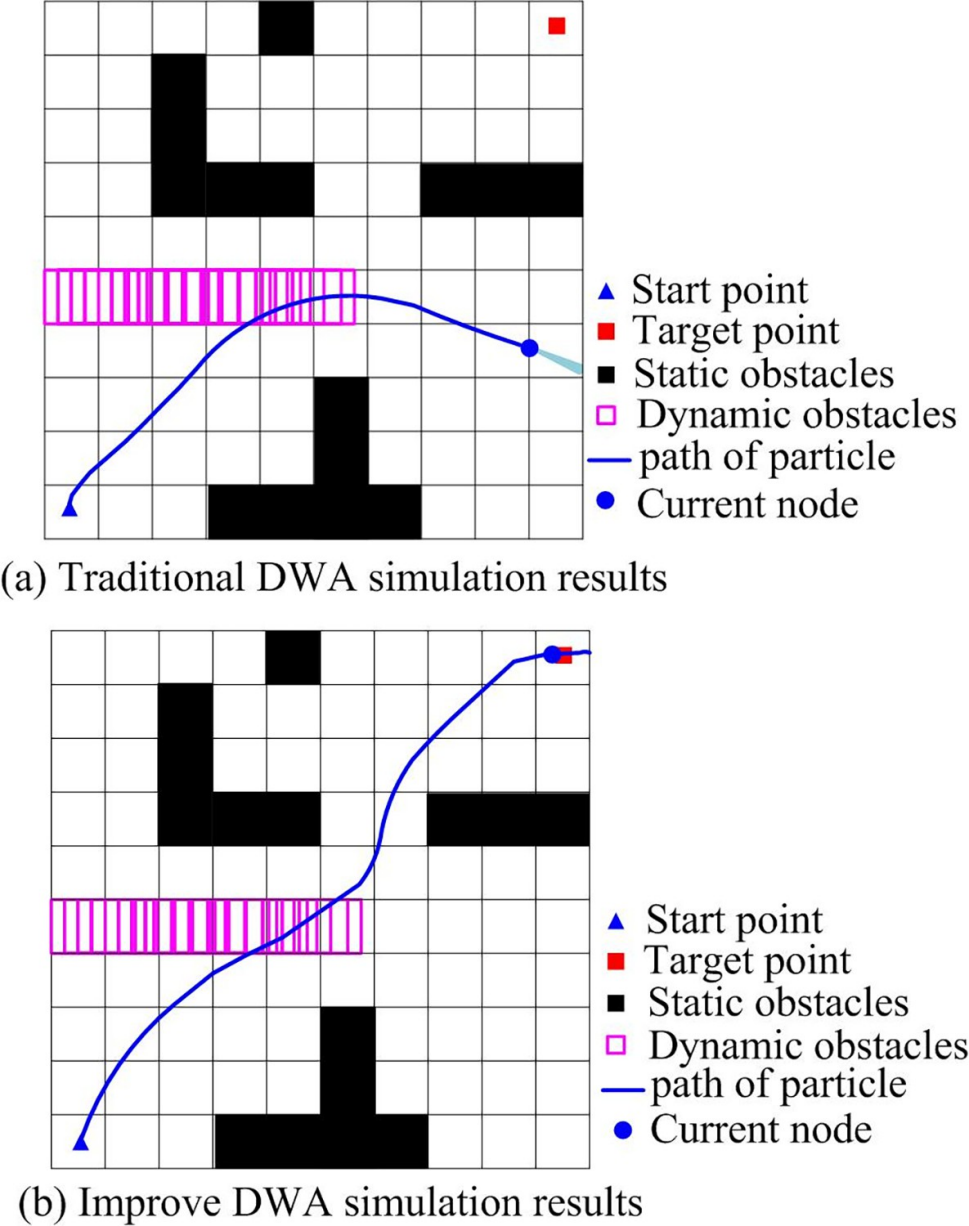
Box plot of experimental results for different algorithms.

In Fig 8(A), in terms of path planning time, median planning time required by GA and SA algorithms is 331.77ms and 151.83ms, which is much larger than that of the improved A* algorithm. The operation efficiency is low, and SA is unstable in path planning. As shown in Fig 9(B), the planned path length of GA and SA algorithms is the same, greater than that smoothed by A* algorithm, that is, A* path smoothness optimization is better than GA and SA. As shown in Fig 8(C), the median number of robot turns of GA and SA algorithms are 34 and 25 respectively, which are higher than those of the improved A* algorithm. To sum up, the performance of GA and SA algorithms is less efficient than A* algorithm, and it takes a lot of time to plan the path, that is, the research on the improved A* algorithm improves algorithm efficiency. Fig 8(D) shows that the corresponding path planning of each algorithm can be viewed in a 30×30 raster map. It can be seen that neither simulated annealing algorithm nor genetic algorithm can achieve obstacle avoidance well, and there are many vertical changes in the path, which is not conducive to the direction change of the robot in practical application, and it is easy to fall and other phenomena. The path obtained by traditional algorithms presents a more tortuous and longer path. The proposed path planning algorithm not only can avoid obstacles, but also its path is relatively smooth, which is conducive to the actual walking of the robot.

### 4.2. Obstacle avoidance path planning results analysis of hybrid robot based on DWA

The research will first carry out simulation experiments on the improved sliding window algorithm, compare and analyze DWA performance before and after improvement, and then further carry out experiments on the hybrid algorithm of global planning using improved A* and local path planning based on improved DWA, and analyze results by evaluating function weight, path planning time and average algorithm running speed. Improved DWA is simulated in grid map with dynamic obstacles below.

**Fig 10:**
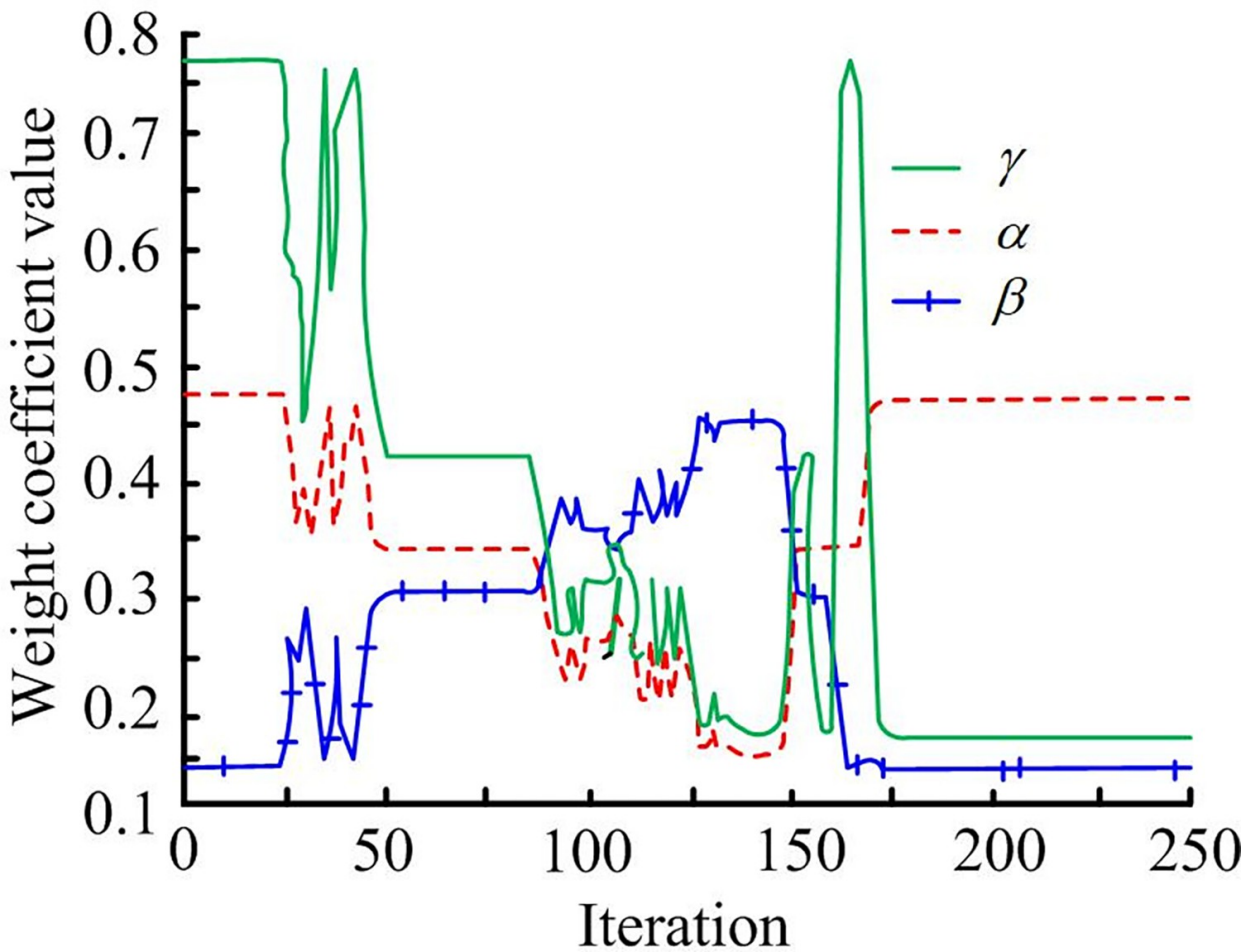
Improving the weight coefficients of DWA algorithm.

Fig 9(A) shows robot trajectory diagram for path planning simulation through traditional DWA. At the beginning, robot moves toward target, but when encountering dynamic obstacles, it deviates from target point to avoid obstacle trajectory and fails to reach the target, which is due to the small weight of the target direction angle. Fig 9(B) shows robot obstacle avoidance roadmap by the improved DWA simulation. Robot successfully avoided dynamic and static obstacles and reached target point. To further analyze local path planning of improved robot, the change graph of the weight of robot evaluation function in algorithm operation represents robot obstacle avoidance. Weight coefficient of improved DWA algorithm is shown in Fig 10.

**Fig 11:**
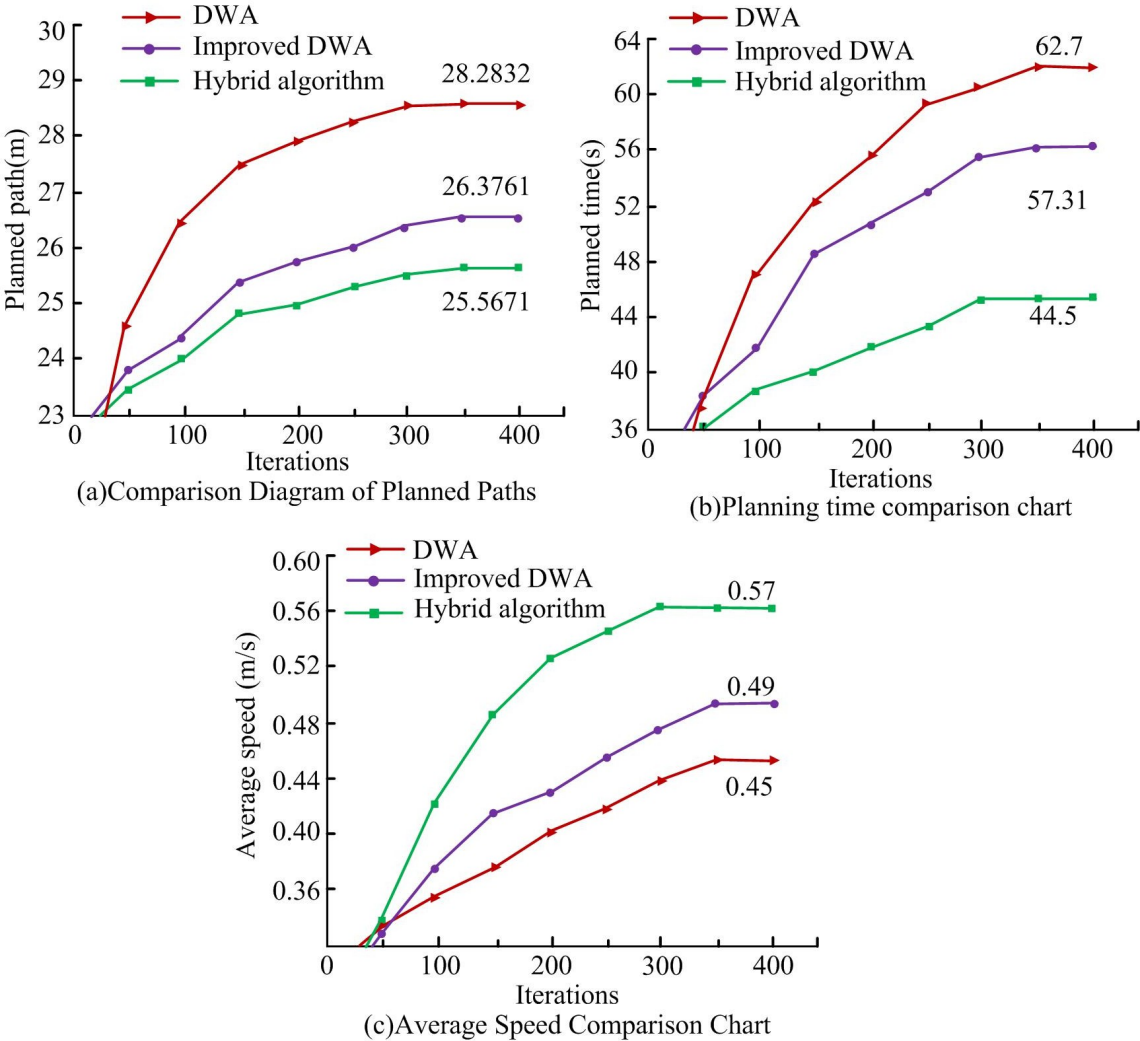
Improving the weight coefficients of DWA algorithm.

As shown in Fig 10, at the beginning, the weight of speed *γ* and azimuth *α* output of fuzzy controller is large, and the weight of obstacle gap *β* is small, indicating that the robot is far away from target point and the obstacle; If iteration reaches 50, *β* starts to increase, *γ* and *α* gradually decrease, indicating that robot starts to approach obstacle, moves towards target and starts to move away from the obstacle; If iteration is between 50 and 140, the weight coefficient response is intense, indicating that the robot is avoiding dynamic obstacles; When the number of iterations is about 140, *β* starts to decrease, *α* and *γ* gradually increase, indicating that robot moves towards target point; When the target point is 250 times, robot reaches successful target point. A comparison is made between the traditional and improved DWA and the hybrid algorithm that combines improved A* and DWA. Experimental results are shown in Fig 11.

**Fig 12:**
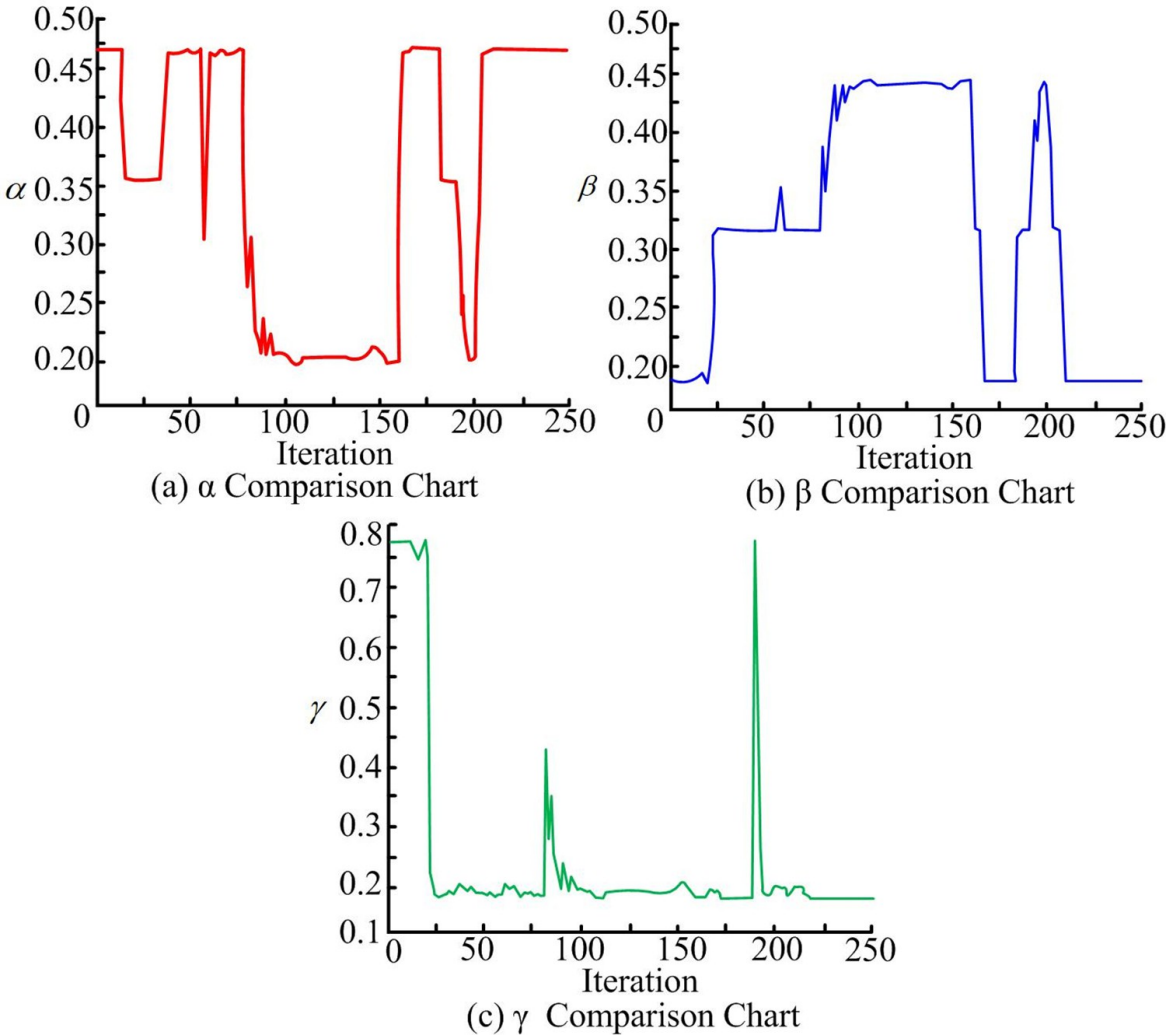
Improve the weight coefficient of DWA evaluation function.

As shown in Fig 11, the hybrid algorithm obtained the shortest planning length and time, which are 25.5671m and 44.5s respectively, and the highest average speed is 0.57m/s. Among them, planning path length of hybrid algorithm decreased by 9.6% compared with the traditional method, and about 3.1% compared with improved DWA; Compared with traditional DWA, the path planning time of hybrid algorithm decreased by 29%, and compared with improved DWA, it is reduced by about 22.4%; Hybrid robot average speed increased by 26.7% compared with the traditional method and about 8.9% compared with the improved method. In general, the hybrid algorithm has the highest efficiency in path planning and has certain advantages. Meanwhile, research will analyze path planning by changing the weight of robot evaluation function. Hybrid weight coefficient is shown in Fig 12.

**Fig 13:**
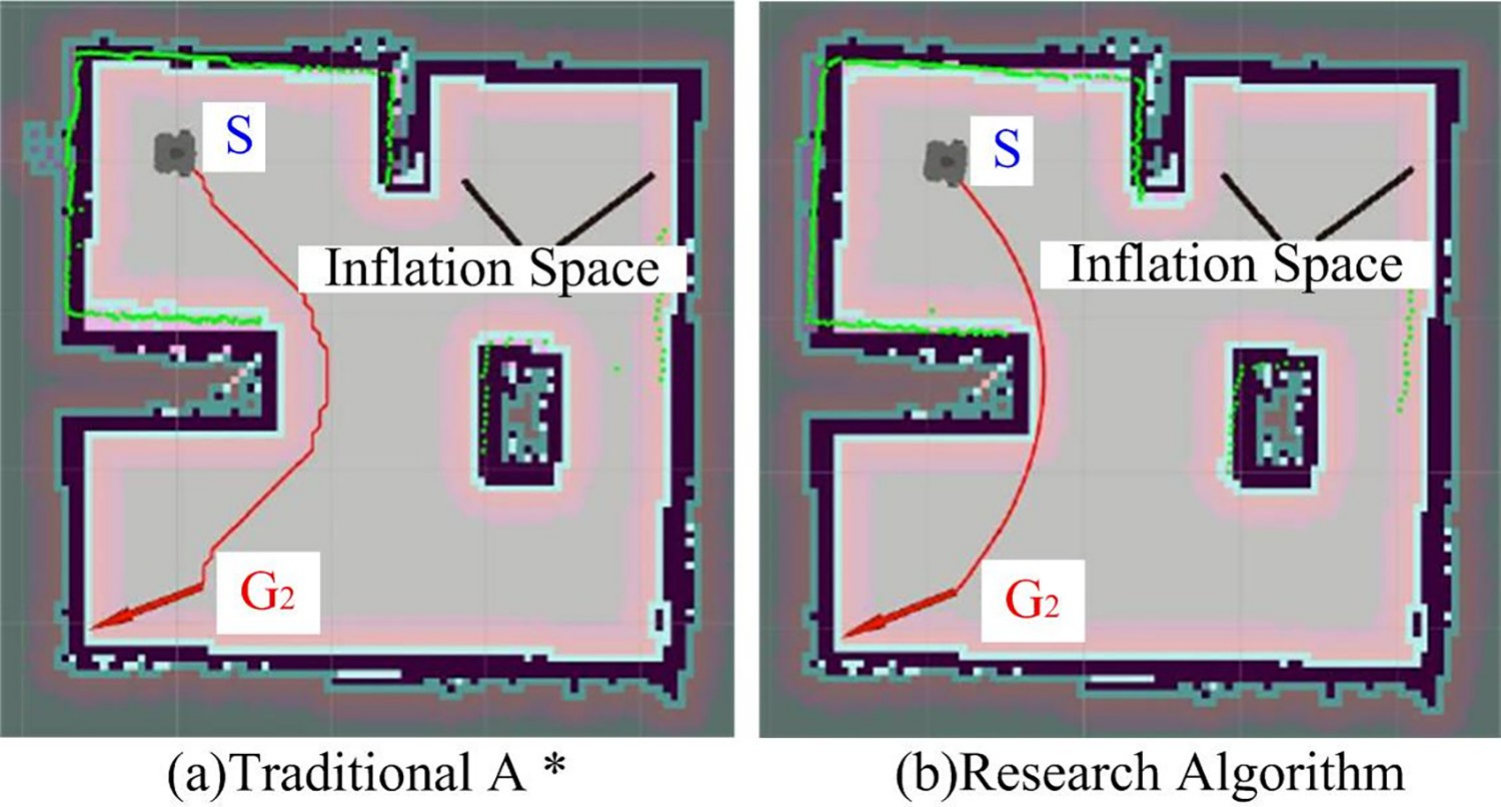
Traditional A* and research algorithm planned paths perform successfully.

In Fig 12, before iteration reaches 180, the weights are relatively stable, and the obstacles encountered are static, *α* decreasing, *β* increasing, and *γ* decreasing indicates encountering obstacles; When the number of iterations is about 180, *α* starts to decrease, *β* and *γ* reach the minimum, indicating that the obstacle is moving at this time. When the program is iterated to 184 times, the robot is closest to the obstacle, which is 1.238m; When the number of iterations reaches about 190, the robot successfully avoids obstacles, the speed increases rapidly, and moves towards the target point. Finally, robot reaches target point. If path planning performance is verified only through software based simulation experiments, the feasibility of the algorithm cannot be directly proved. The research will build a real scene map, introduce the turtlebot3 robot pair, scan the actual scene and establish the corresponding grid map for experiments, that is, verify algorithm feasibility through real scene experiments, as shown in Fig 13.

However, the obstacle avoidance path planned by traditional A* has turned many times, and the planned route is rugged, not smooth, and the planned path is longer. Path planned by research algorithm is relatively smooth, and overall path and time to reach target point are shorter, indicating that research algorithm has higher path planning efficiency in practical application. In order to further verify the reliability of the proposed A*-DWA algorithm, HFAMCPSO and RRT*-PSO algorithms were introduced to conduct a control experiment, and the experimental scene was also from the map in the corresponding literature [23]. The experimental results are shown in Table 3.

**Table 3:** Comparison of experimental data of different algorithms in different maps.

Map	Algorithm	Index	Data
[600×800] cm	HFAMCPSO	Path planning length	737.399cm
	A*-DWA		720.145cm
	HFAMCPSO	Number of iterations	50
	A*-DWA		42
[500×500] cm	RRT*-PSO	Path planning length	615.30cm
	A*-DWA		580.42cm
	RRT*-PSO	No. of input node	14
	A*-DWA		11

As can be seen from Table 3 above, in the map of [600×800] cm, the performance of the two comparison algorithms is relatively excellent, but the proposed A*-DWA algorithm achieves better results with certain advantages. The length of the optimal path and the number of iterations to obtain the path are 2.4% and 19.04% lower than the HFAMCPSO algorithm, respectively. In the [500×500] cm map, the optimal path of the A*-DWA algorithm is 6.01% lower than that of the RRT*-PSO algorithm, and the optimal path of the No. input node is 27.27% lower than that of the RRT*-PSO algorithm. In conclusion, the proposed A*-DWA algorithm can plan the best path in a shorter time.

## 5. Discussion

A robot trajectory obstacle avoidance method based on improved A * and DWA algorithm fusion algorithm was proposed in the study, and this method was verified through experiments. Simulate the improved A * and compare it with the traditional A *. As the grid size increases, the reduction in path planning time of the improved algorithm also increases. The improved algorithm also shows significant improvements in smoothing path length and other aspects. The performance efficiency of genetic algorithm and SA algorithm is lower than that of A * algorithm, and path planning requires a lot of time. Therefore, the research on the improved A * algorithm has improved the efficiency of the algorithm. Conduct simulation experiments on the improved sliding window algorithm, compare and analyze the DWA performance before and after the improvement, and then conduct experiments on the improved A * and the global planning hybrid algorithm based on the improved DWA local path planning, and evaluate the weight of the function. Robot obstacle avoidance roadmap obtained through improved DWA simulation. The robot successfully avoids dynamic and static obstacles and reaches the target point. Further analyze and improve the local path planning of robots, and the weight change graph of the robot evaluation function in algorithm operation represents robot obstacle avoidance. The shortest planning length and time obtained by the A * and DWA hybrid algorithm, the path planned by the research algorithm is relatively stable, and the overall path and time to reach the target point are relatively short, indicating that the research algorithm has high path planning efficiency in practical applications. It can be seen that the proposed A *—DWA algorithm can quickly determine the optimal path. The innovation of this method lies in the combination of global planning and local planning, which solves the problem of path planning failure caused by sudden situations in a short time, and realizes the robot’s obstacle avoidance more efficient and accurate. The proposed method has wide application potential, especially in the field of automation and robot navigation, which require fast response and high-precision path planning, and can significantly improve the autonomy and efficiency of robots in unknown or changing environments.

## 6. Conclusion

At present, the research of robot path planning only adopts global or local methods, which is difficult to satisfy real-time and integrity in complex and changeable environment. This study will optimize the evaluation function, sub node selection method and path smoothness of traditional A* algorithm, introduce fuzzy control to optimize traditional DWA, and design a hybrid method by improved A* and DWA to solve existing problems of obstacle avoidance path planning. The results show that, firstly, improved A* algorithm has a better performance at 30 ×30 grid map, the smoothed path length is reduced by about 16%, path planning time is reduced by nearly 21%, and the number of turns is reduced by about 43%, indicating that the improved algorithm has obvious improvements in the smooth path length, the number of turns and path planning time. Secondly, through path planning simulation experiment of DWA algorithm before and after the improvement, after dynamic obstacles introduction, improved algorithm planning path successfully reached target point, while the traditional method deviated from the trajectory and did not reach target point. When the program is iterated to 184 times, the robot is closest to the obstacle, which is 1.238m; When the number of iterations reaches about 190, the robot successfully avoids obstacles, the speed increases rapidly, and moves towards the target point. Finally, robot reaches target point. Finally, compared with traditional DWA and improved DWA, planning path length of the hybrid algorithm decreased by 9.6% compared with the traditional method, and about 3.1% compared with the improved DWA method; Compared with DWA, path planning time decreased by 29% and 22.4%; Average speed of the robot is 26.7% higher than that of the DWA method and 8.9% higher than that of the improved method. In general, the hybrid algorithm has the highest efficiency of path planning, and addresses the limitation of traditional algorithms by effectively avoiding dynamic obstacles and demonstrating strong adaptability, which has certain advantages. At the same time, the dynamic obstacles in this study are in low-speed motion state, and there is still the possibility of collision in the face of high-speed moving obstacles. In the future, we can study the emergency obstacle avoidance path planning of high-speed obstacles. In terms of positioning and path planning for lawn mowing robots, UWB base stations combined with differential methods can also be considered for implementation. In the future, this scheme can be compared with the positioning and path planning methods in this article to select a better one. And in the path planning process, real-time progress display on the PC side can also be considered, which can allow users to have a clearer understanding of the working status of the lawn mower robot, gradually transitioning from single unit to multi machine collaborative operation.

The significance of this study is that the proposed hybrid path planning algorithm significantly improves the obstacle avoidance ability and path planning efficiency of robots in dynamic environments. This algorithm optimizes the traditional A* and DWA algorithms, enabling robots to realize fast and efficient path planning in more complex environments, and provides innovative technical support for the development of automated robot assistance systems.

## Supporting information

S1 File (ZIP)
