# Supplementary figures and images for: Robot obstacle avoidance optimization by A* and DWA fusion algorithm
Source: PLoS One. 2024 Apr 29;19(4):e0302026. doi: 10.1371/journal.pone.0302026 (PMC11057753; doi:10.1371/journal.pone.0302026)

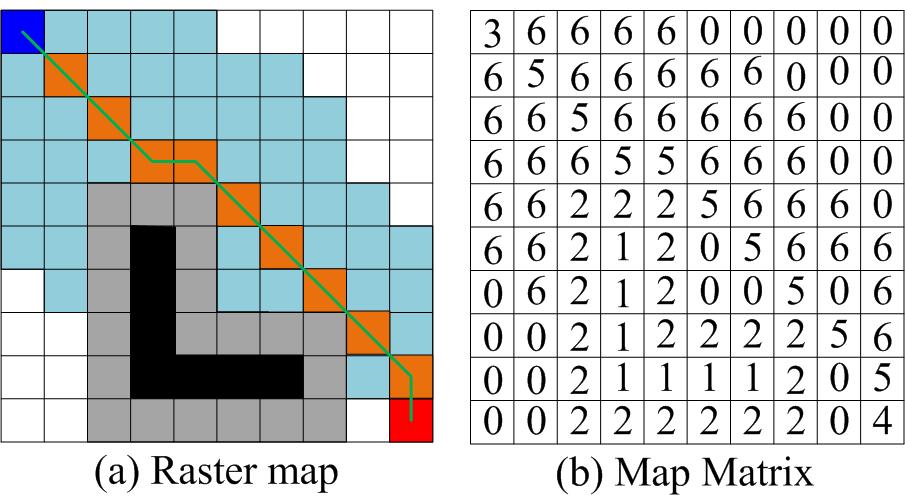

Supplement: S1 File — (ZIP) [file pone.0302026.s001.zip › Supporting information/Figure 1.jpg]

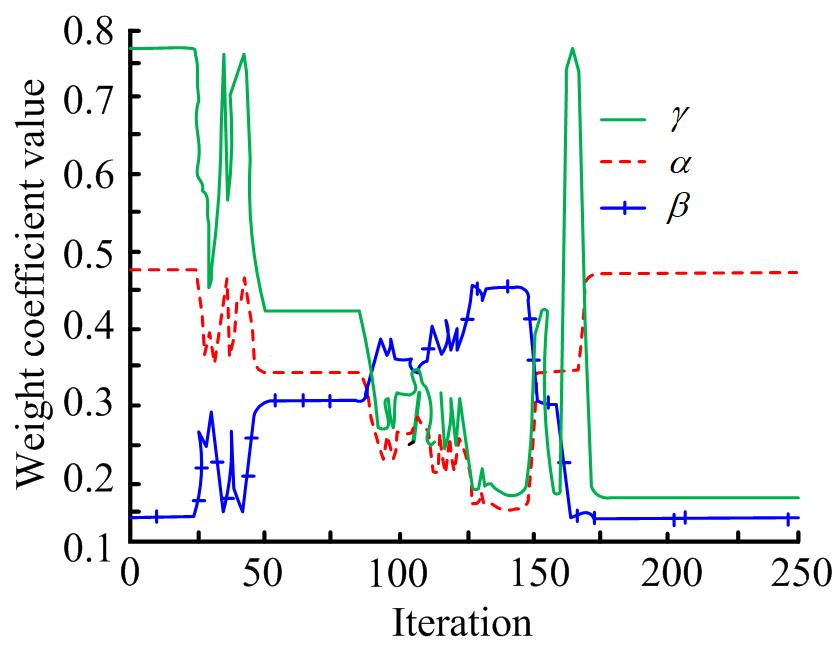

Supplement: S1 File — (ZIP) [file pone.0302026.s001.zip › Supporting information/Figure 10.jpg]

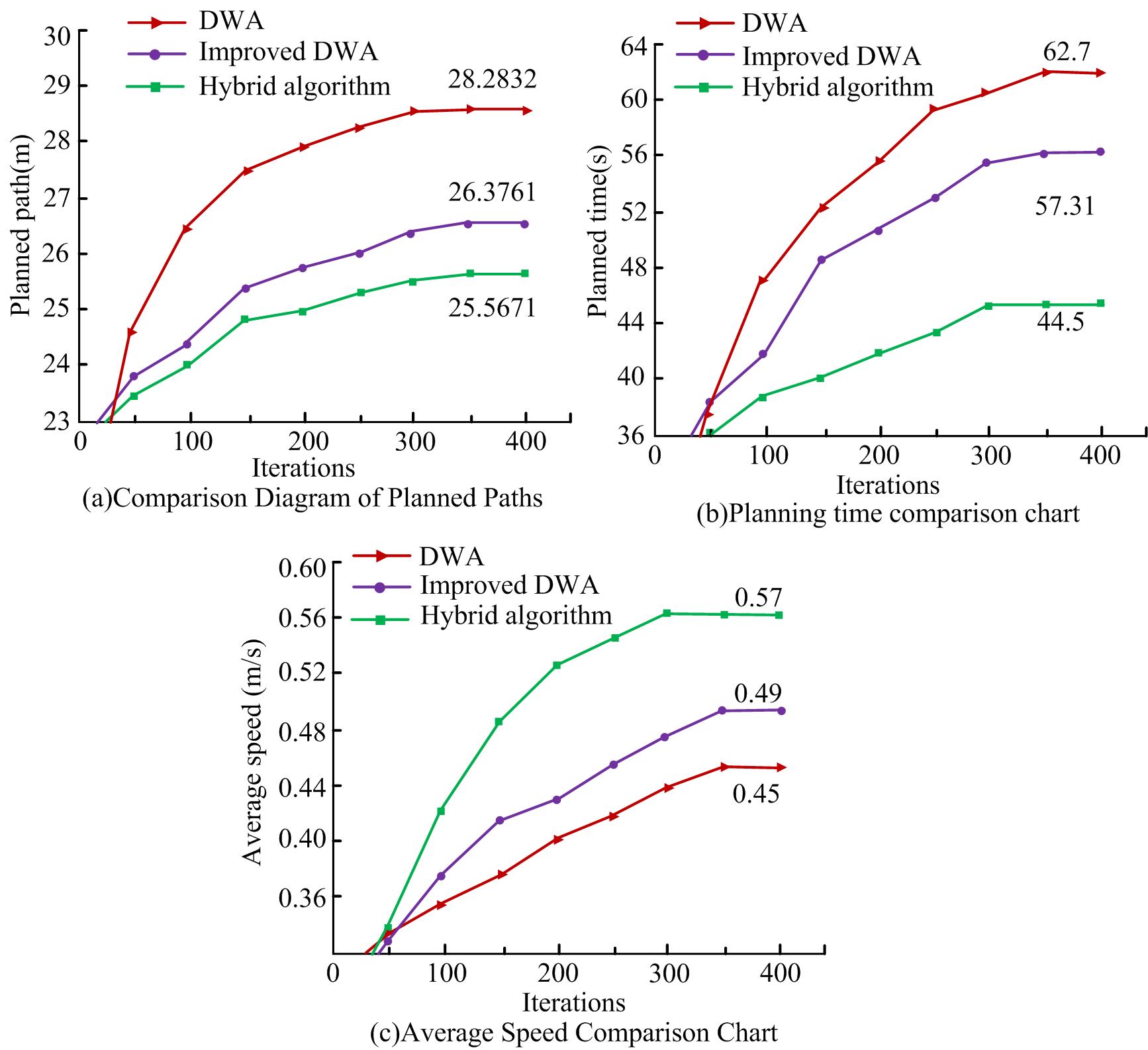

Supplement: S1 File — (ZIP) [file pone.0302026.s001.zip › Supporting information/Figure 11.jpg]

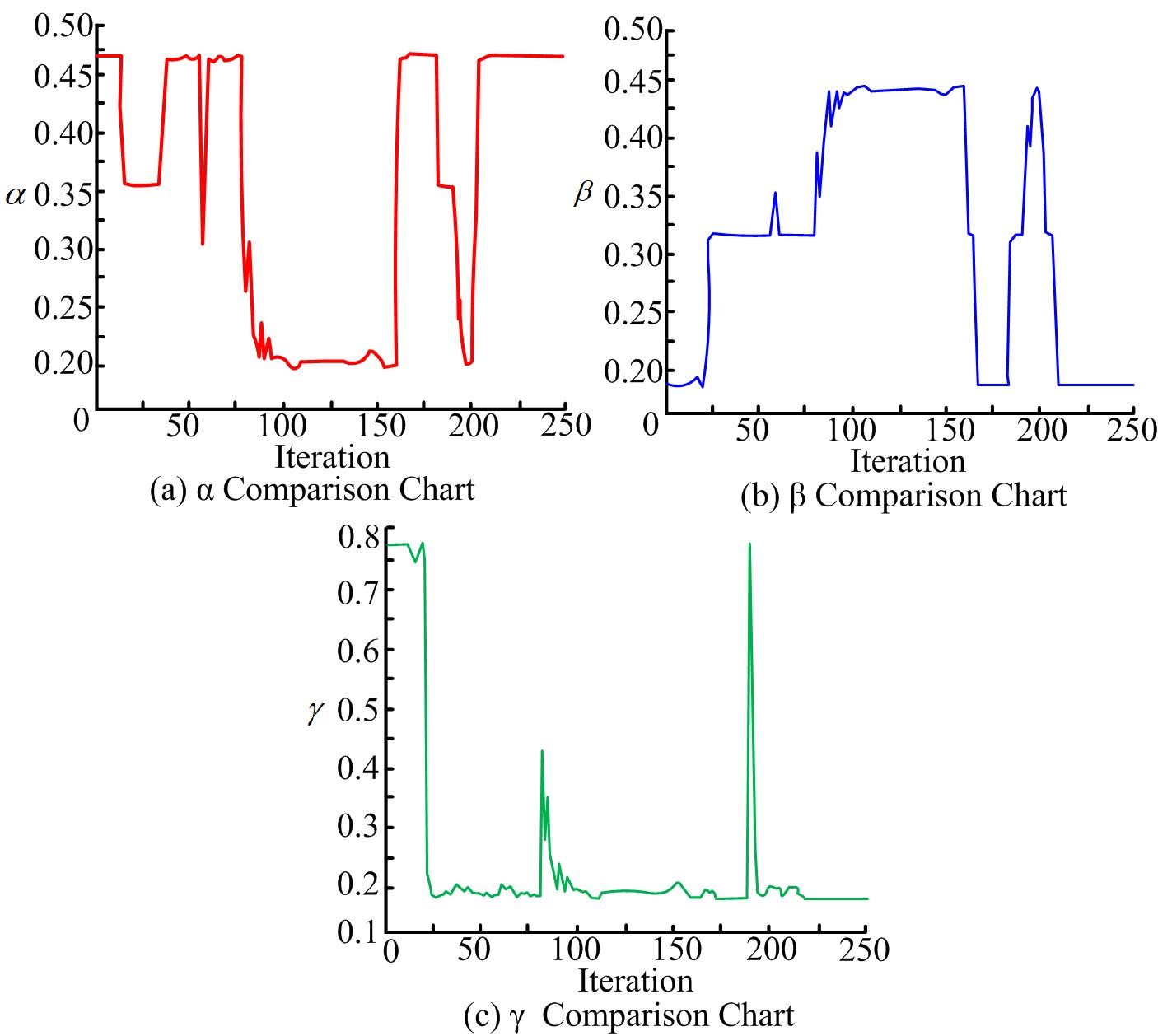

Supplement: S1 File — (ZIP) [file pone.0302026.s001.zip › Supporting information/Figure 12.jpg]

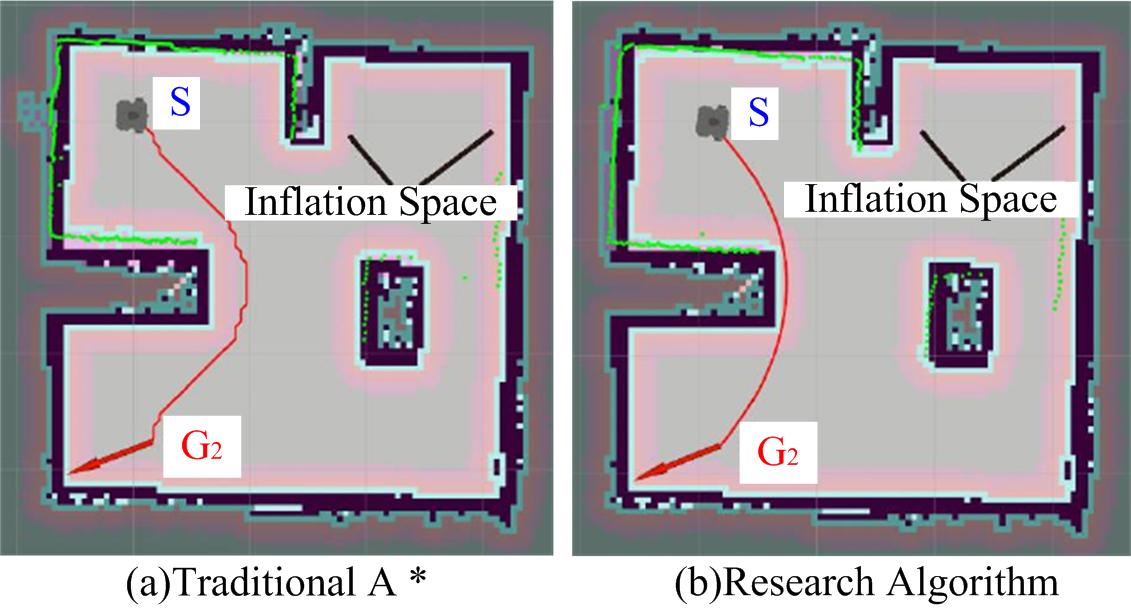

Supplement: S1 File — (ZIP) [file pone.0302026.s001.zip › Supporting information/Figure 13.jpg]

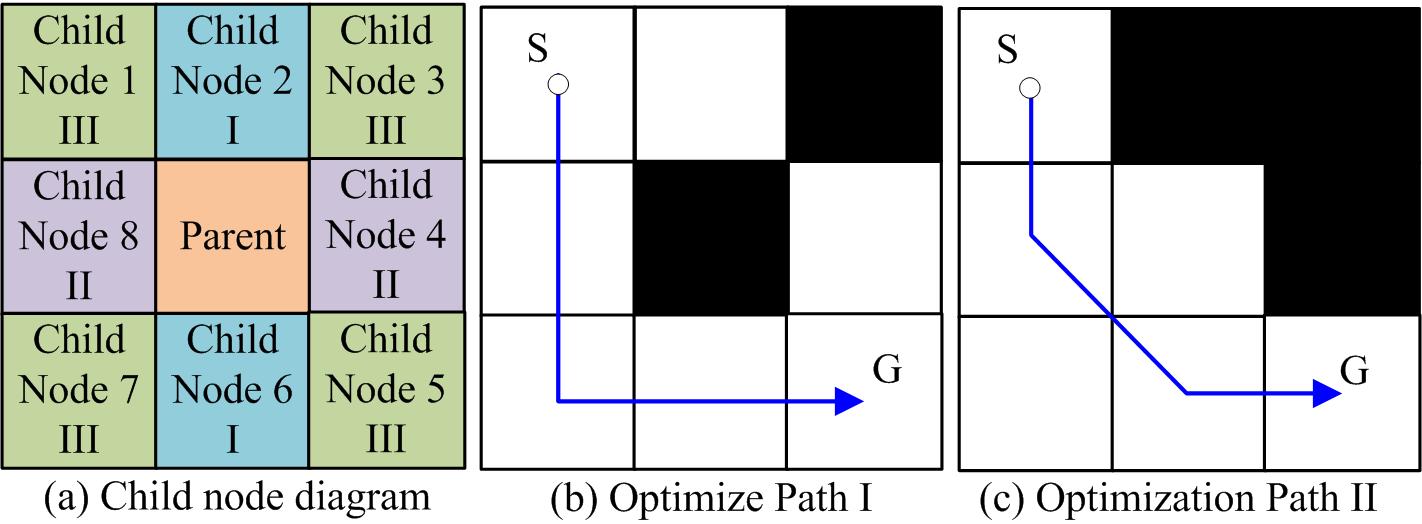

Supplement: S1 File — (ZIP) [file pone.0302026.s001.zip › Supporting information/Figure 2.jpg]

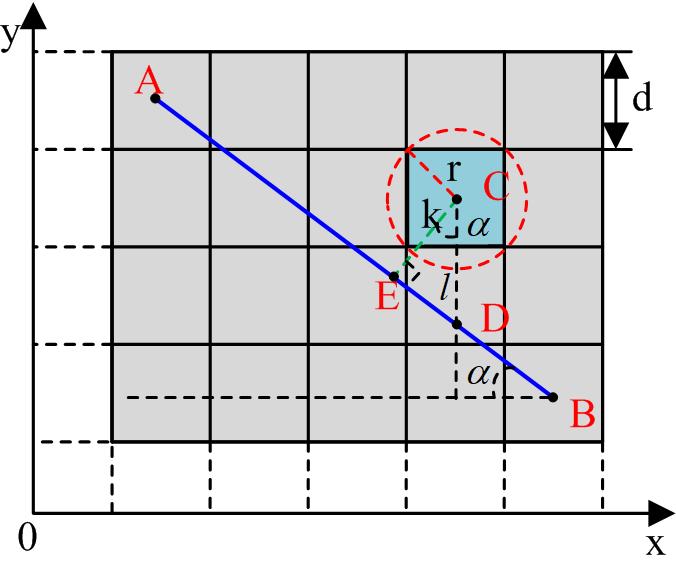

Supplement: S1 File — (ZIP) [file pone.0302026.s001.zip › Supporting information/Figure 3.jpg]

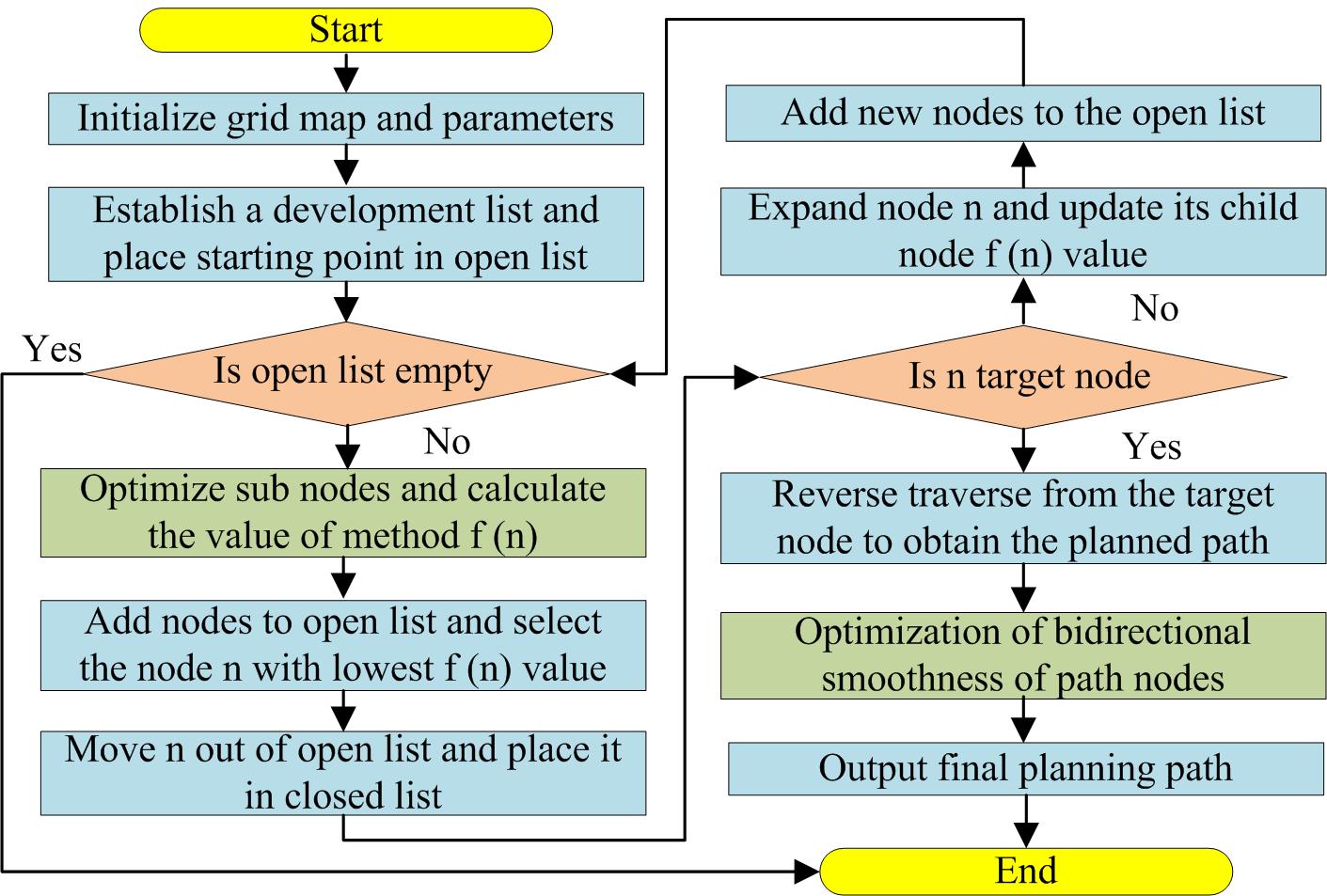

Supplement: S1 File — (ZIP) [file pone.0302026.s001.zip › Supporting information/Figure 4.jpg]

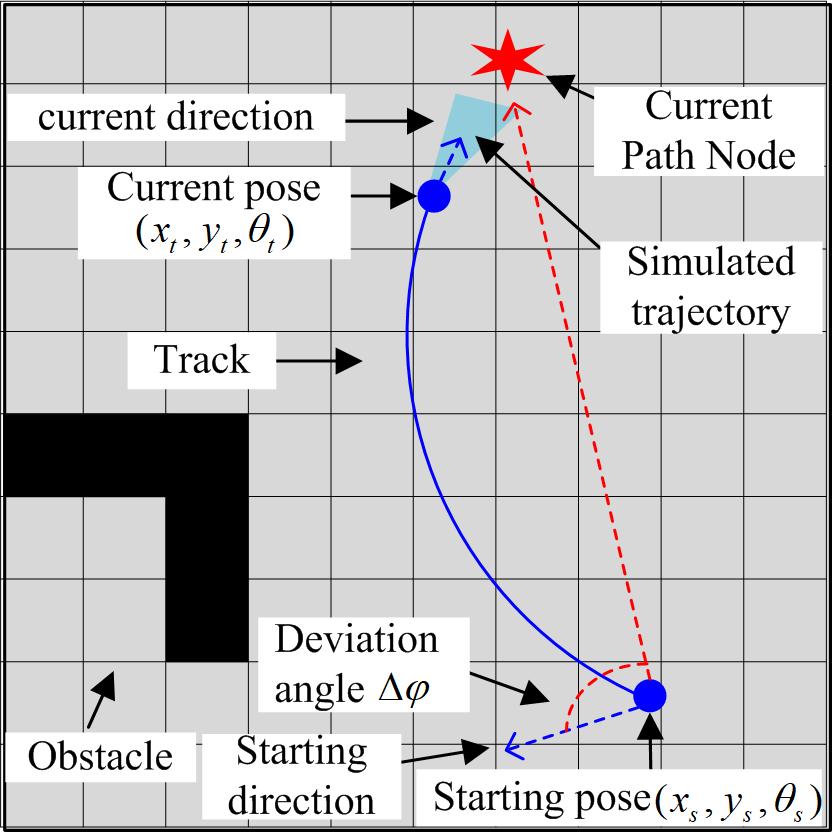

Supplement: S1 File — (ZIP) [file pone.0302026.s001.zip › Supporting information/Figure 5.jpg]

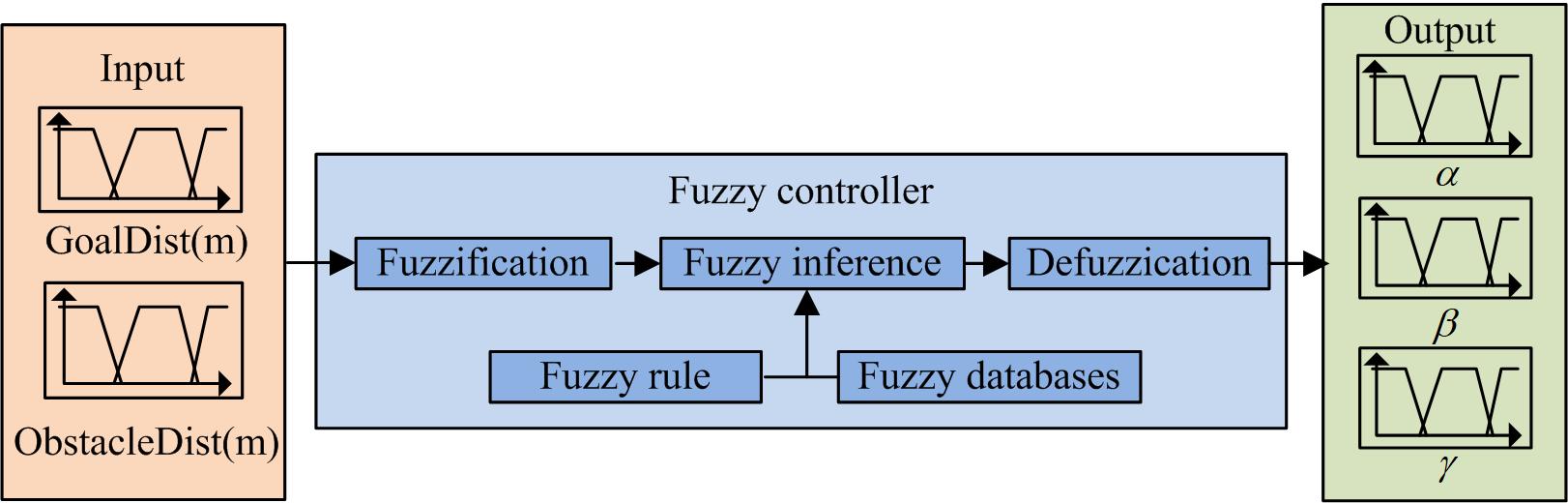

Supplement: S1 File — (ZIP) [file pone.0302026.s001.zip › Supporting information/Figure 6.jpg]

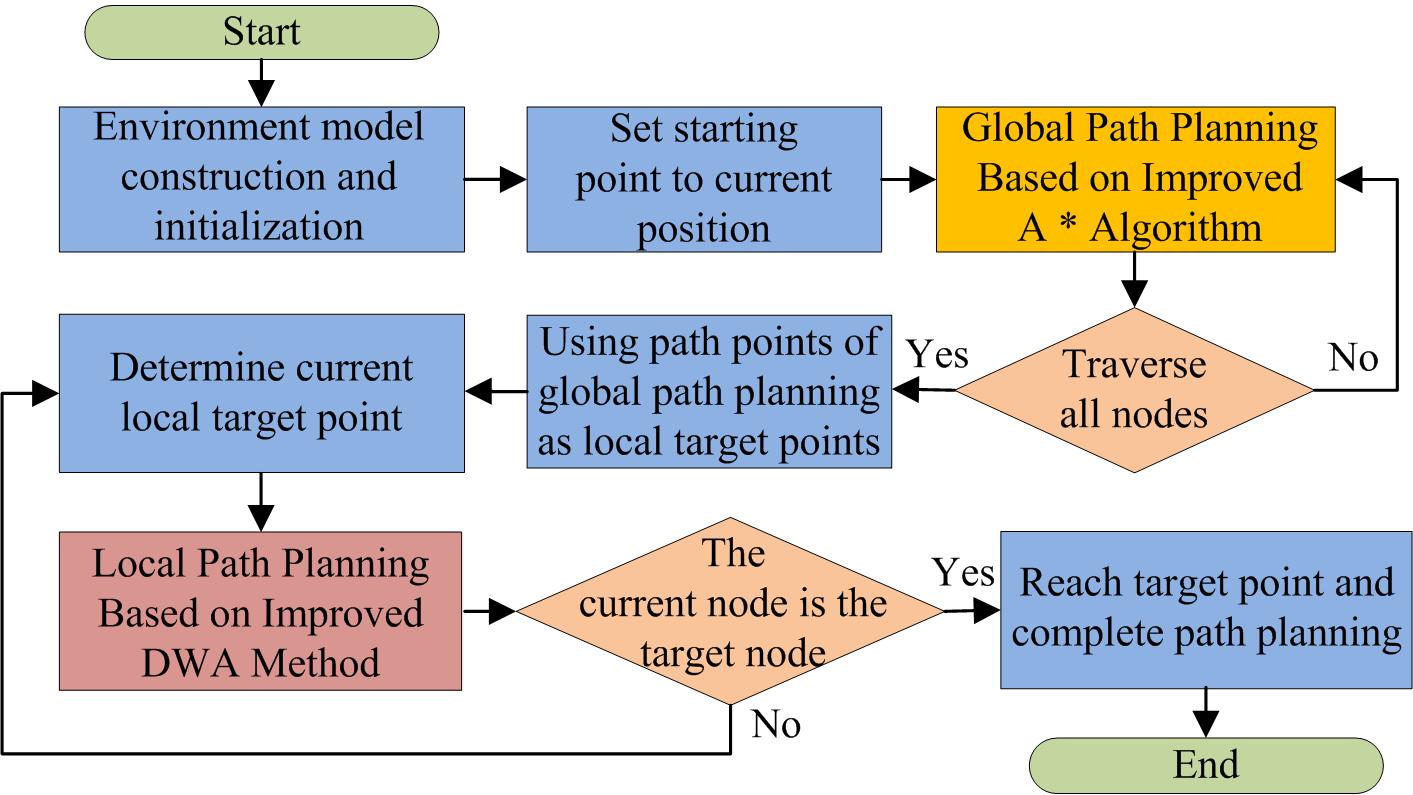

Supplement: S1 File — (ZIP) [file pone.0302026.s001.zip › Supporting information/Figure 7.jpg]

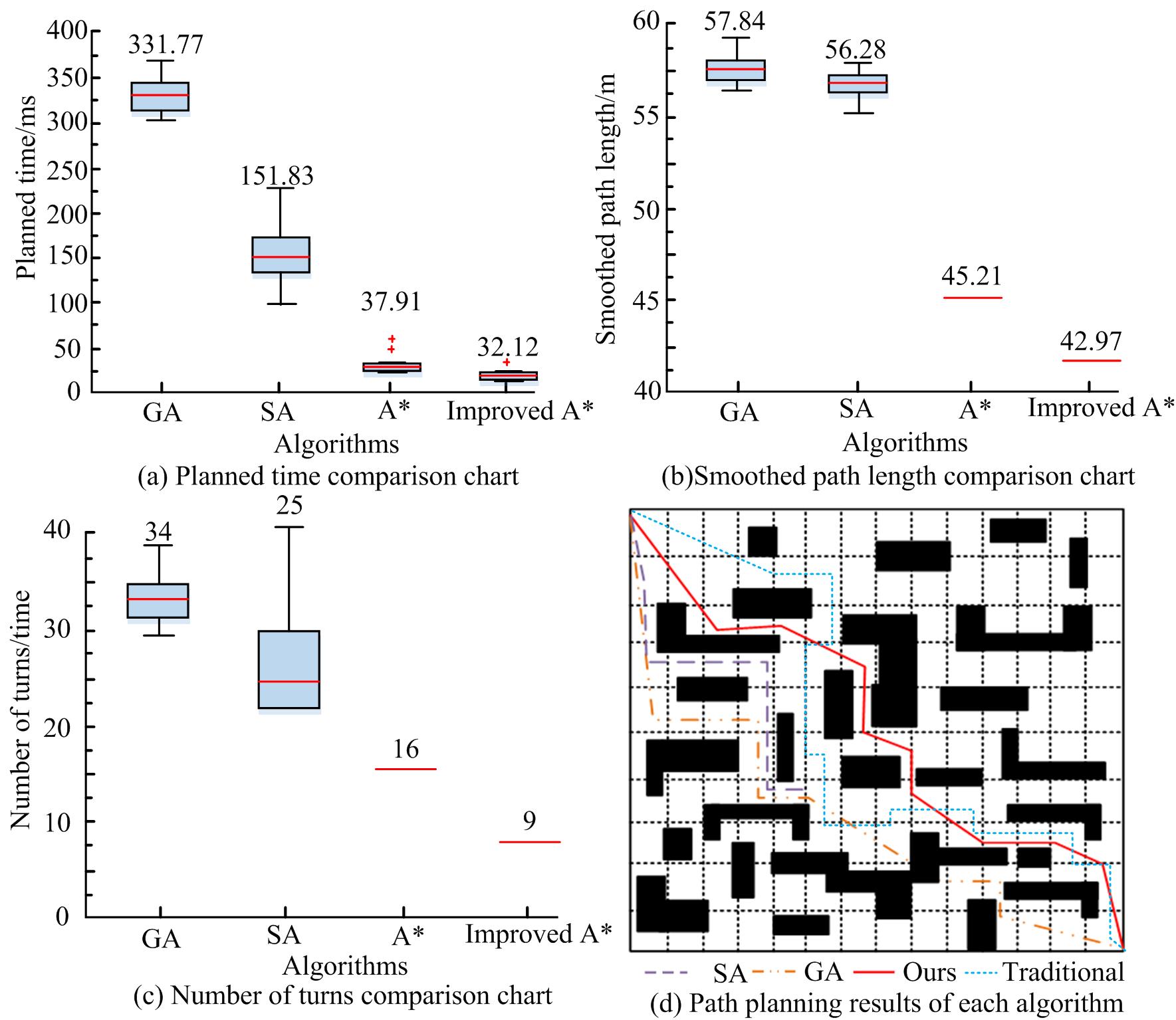

Supplement: S1 File — (ZIP) [file pone.0302026.s001.zip › Supporting information/Figure 8.jpg]

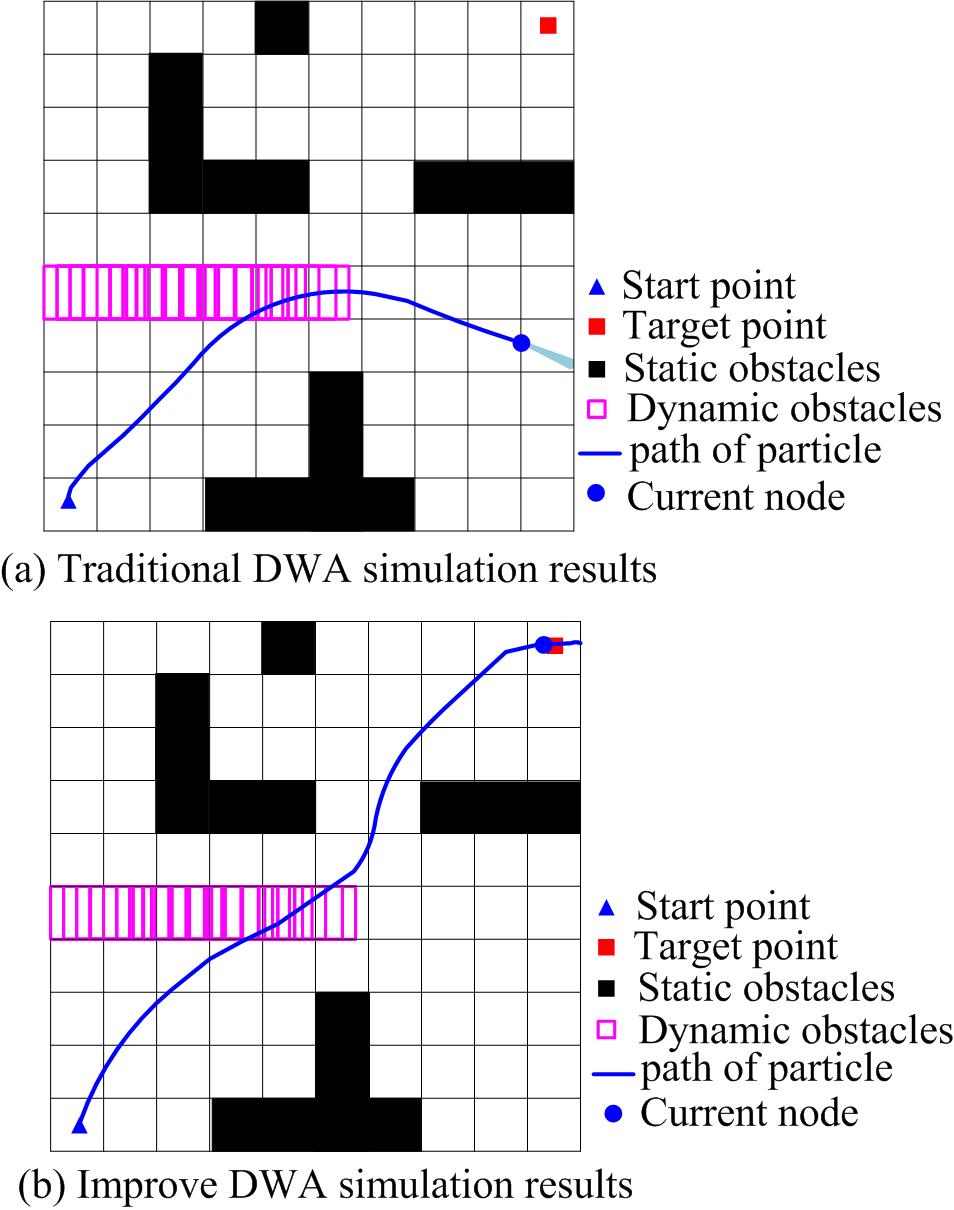

Supplement: S1 File — (ZIP) [file pone.0302026.s001.zip › Supporting information/Figure 9.jpg]
